# NAD^+^ augmentation by nicotinamide riboside engages SLIT2/ROBO1 signaling to attenuate Th17 inflammation in psoriasis

**DOI:** 10.1172/jci.insight.203826

**Published:** 2026-04-28

**Authors:** Kim Han, Rachael J. Klein, Thomas C. Recupero, Anna Chiara Russo, Rahul Sharma, Anand K. Gupta, Shahin Hassanzadeh, Rebecca D. Huffstutler, Pradeep K. Dagur, Bryan Fisk, Neelam R. Redekar, Michael N. Sack

**Affiliations:** 1 Laboratory of Mitochondrial Biology and Metabolism, NHLBI,; 2 Cardiovascular Branch, NHLBI,; 3 Flow Cytometry Core Facility, NHLBI, and; 4 Integrated Data Science Section, NIAID, NIH, Bethesda, Maryland, USA.

**Keywords:** Dermatology, Immunology, Adaptive immunity, Clinical trials, Signal transduction

## Abstract

**BACKGROUND:**

Enhancing NAD^+^ levels with nicotinamide riboside (NR) confers antiinflammatory effects in human disease, although immunoregulatory mechanisms remain poorly characterized. We previously showed that ex vivo NR supplementation of primary CD4^+^ T cells from psoriatic individuals dampened immune responsiveness.

**METHODS:**

To validate this in vivo, we performed a randomized, placebo-controlled NR supplementation study in individuals with mild-to-moderate psoriasis. Participants received oral NR (500 mg twice daily) or matching placebo for 4 weeks, with blood samples collected at baseline and after supplementation. NR reduced Th17 immune responsiveness.

**RESULTS:**

Bulk CD4^+^ T cell RNA-seq identified induction of the SLIT-ROBO signaling pathway. NR supplementation increased circulating SLIT2 levels and enhanced SLIT2 production in dermal fibroblasts. Pharmacologic and genetic interrogation in CD4^+^ T cells and fibroblasts demonstrated that SLIT2, acting through the ROBO1 receptor, inhibited Rho GTPase signaling, thereby attenuating canonical Th17 polarization and fibroblast inflammatory activation.

**CONCLUSION:**

These findings indicate that NAD^+^ augmentation exerts anti-inflammatory effects in psoriasis through SLIT2-ROBO1-mediated crosstalk between dermal fibroblasts and circulating CD4^+^ T cells, leading to suppression of Th17-driven inflammation.

**TRIAL REGISTRATION:**

ClinicalTrials.gov NCT04271735 (registration date – 2020-08026), NCT01143454 (registration date - 2010-07-21), NCT01778569 (registration date – 2013-01-22), and NCT00001846 (registration date – 2001-01-11).

**FUNDING:**

The NHLBI Division of Intramural Research (HL005102 – MNS).

## Abstract 2

Specific vitamin B3 supplements have anti-inflammatory effects. This paper begins to explore how this works to blunt skin and blood inflammation in psoriasis.

## Graphical abstract

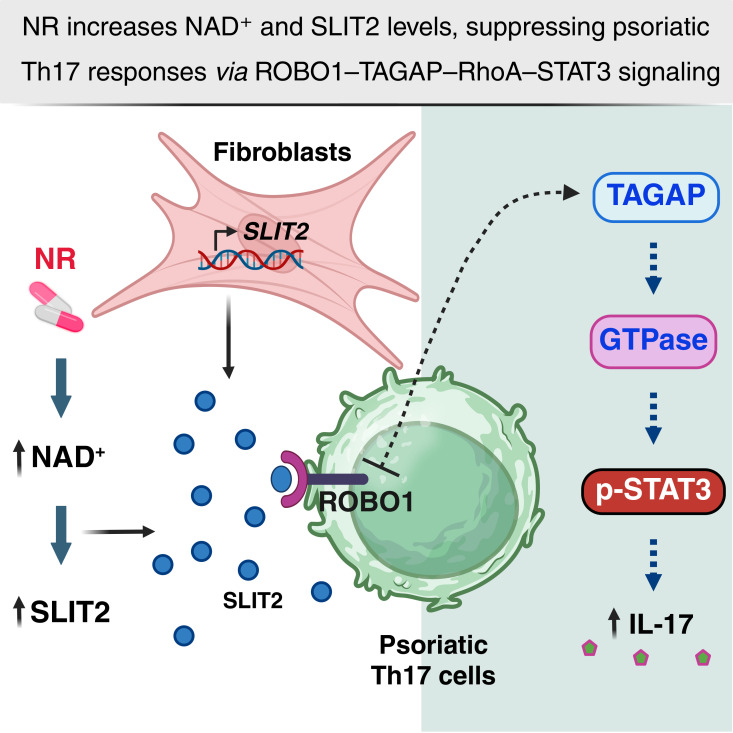

## Introduction

Nicotinamide adenine dinucleotide (NAD) enables key redox reactions that generate ATP, which is vital for eukaryotic cell metabolism. Beyond energy production, NAD^+^ is an essential signaling molecule and substrate for enzymes that coordinate an array of processes from DNA repair to metabolic reprogramming. Ongoing genetic, biochemical, and physiologic murine studies are defining the complexity and redundancy in NAD^+^ biosynthesis and salvage pathways (1–3). This biochemistry occurs within specific organs, between organs and the gut microbiome (4–6). Furthermore, genetic mitochondrial defects, degenerative, autoimmune, and inflammatory diseases, as well as aging, are all associated with reduced NAD^+^ levels (7–10). This, in turn, is postulated to be linked to the underlying pathophysiology of these diseases and to cellular senescence. The complexity of this biology is underscored by the fact that supplementation with NAD^+^ precursors can have effects ranging from minimal to significant in different human diseases (3, 11). Despite these variables, a consistent effect of NAD^+^ boosting in humans remains the blunting of inflammation (12), although the mechanisms underpinning this remain poorly characterized. Our laboratory explores the immune effects of NAD^+^ boosting using the salvage pathway precursor nicotinamide riboside (NR). We have shown that oral NR supplementation in healthy volunteers blunts both innate and adaptive immune responsiveness (9, 13). In both aging and disease models, other investigators have shown that oral NR supplementation broadly reduces inflammation in degenerative diseases, including heart failure (14) and Parkinson’s disease (8), and during aging (15). In those studies inflammatory signatures in peripheral blood mononuclear cells (14), cerebrospinal fluid (8), and serum (8, 15) were attenuated by NR. In ex vivo studies using primary cells from participants with inflammatory (psoriasis) and autoimmune (systemic lupus erythematosus [SLE]) diseases, we have previously shown that NR blunts CD4^+^ Th1 and Th17 immune responsiveness in psoriasis and monocytic type I IFN activation in SLE (9, 13). These immunomodulatory effects appear to be regulated via NAD^+^-dependent control of distinct metabolic pathways, including effects on inosine and arginine metabolism and downstream intracellular signaling events. To determine whether this immunoregulation is operational in vivo and to advance our understanding of the mechanisms underpinning these NR’s antiinflammatory effects, we performed a 4-week placebo-controlled oral supplementation study in psoriasis participants, with a targeted focus on CD4^+^ T cell biology.

Bulk RNA-seq analysis in CD4^+^ T cells implicated that NAD^+^ boosting may modulate the SLIT roundabout receptor (ROBO) signaling pathway. The SLIT family encodes extracellular membrane-associated glycoproteins that function as ligands for the ROBO family of repulsive guidance cell-surface receptors. The role of this ligand-receptor pathway in immunobiology is currently limited, although, interestingly, the SLIT2/ROBO1 pathway has been shown to modulate transformed human Jurkat T cell chemotaxis (16). We quantified SLIT2 levels in the sera of control versus psoriasis volunteers, and in the psoriasis participants in the placebo versus NR supplementation study. Compared with controls, circulating SLIT2 levels were significantly lower in psoriasis, and NR supplementation increased circulating levels in psoriasis participants.

Considering these finding, we elected to pursue the biology of this pathway in CD4^+^ T cells. In this study, we show that SLIT2 mimicked NAD^+^ boosting by blunting Th17 immune responsiveness and that NR concordantly induced ROBO1 expression in Th17 cells. Molecular loss-of-function studies in primary human CD4^+^ T cells demonstrated that SLIT2/ROBO1 signaling, via inhibition of the T cell Activation Rho GTPase-Activating Protein (TAGAP), dampened canonical inflammatory Th17 cell activation. Furthermore, given that skin fibroblasts are upstream drivers of systemic inflammation in psoriasis (17), we explored the role of NR and SLIT2 in primary skin fibroblasts extracted from a distinct cohort of participants in the control and psoriasis groups. NR supplementation increased SLIT2 release from primary human fibroblasts derived from both healthy control and psoriasis-lesion skin biopsies. Moreover, primary fibroblasts from psoriasis-lesion biopsies showed higher levels of IL-6 secretion, which were blunted by coculture with either NR or SLIT2. Together these findings support a broader role for the SLIT2/ROBO1-RhoGTPase signaling pathway in mediating NAD^+^ boosting antiinflammatory effects in psoriasis and identify ROBO1 as a cell surface receptor that may serve as a putative therapeutic target for this disease.

## Results

### Oral NR attenuates Th1 and Th17 immune responsiveness in psoriasis.

**Figure 1:**
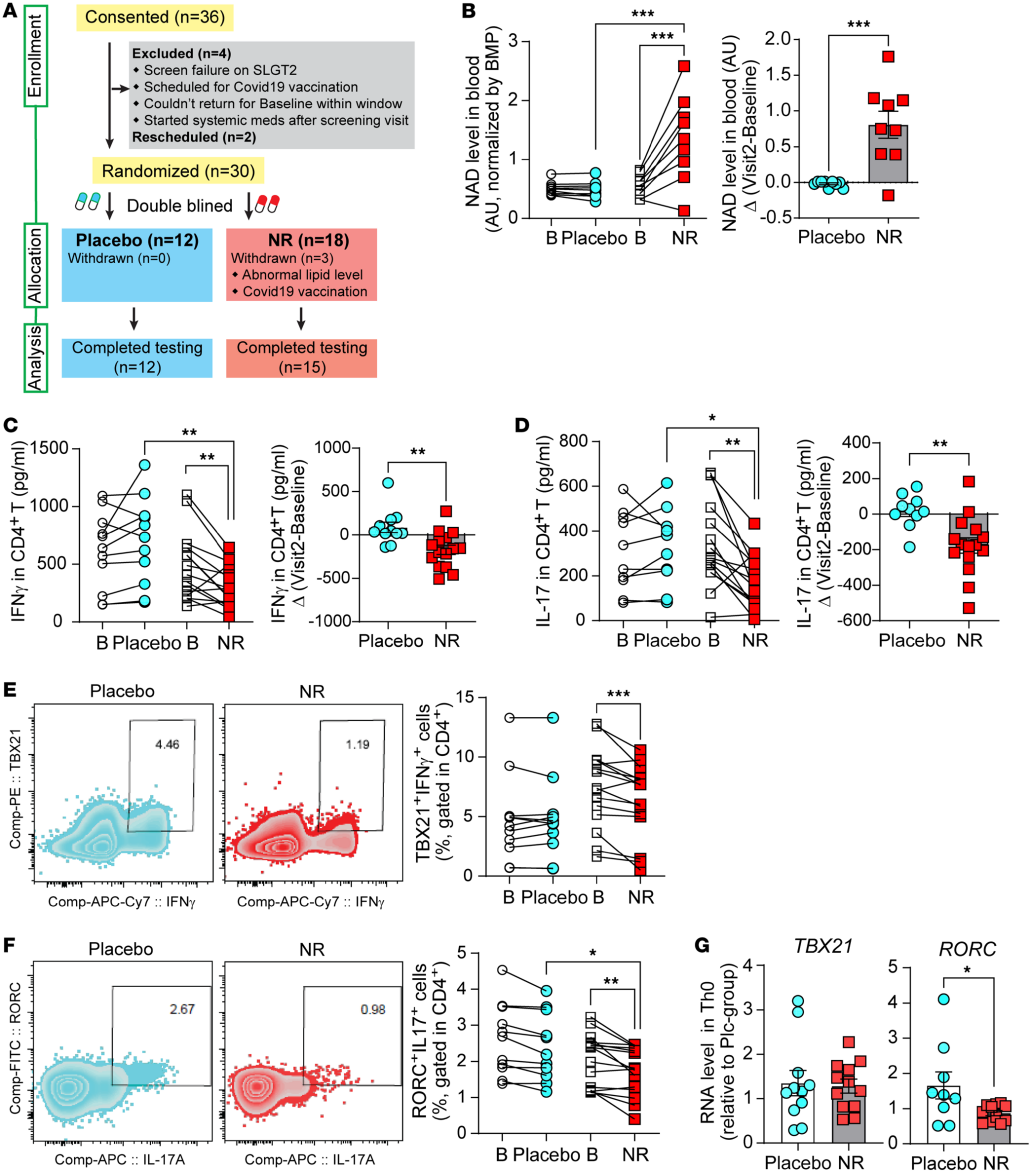
NR supplementation attenuates T cell immune responsiveness. (**A**) CONSORT flow diagram of participant enrollment, allocation, and analysis in the NR supplementation study (ClinicalTrials.gov Identifier: NCT04271735). (**B**) Whole blood NAD^+^ levels by LC-MS using BMP (8-bromoadenosine 5′-monophosphate) as an internal standard (*n* = 9–10/group). Data are presented as the change (Δ = visit 2 − baseline) in NAD^+^ levels. A.U., arbitrary units. (**C** and **D**) IFN-γ and IL-17 secretion from activated CD4^+^ T cells (placebo, *n* = 11; NR, *n* = 15). (**E** and **F**) Flow cytometry of Th1 (IFNγ^+^TBX21^+^) and Th17 (IL-17^+^RORC^+^) populations in live CD45^+^CD3^+^CD8–CD4^+^ T cells. (**G**) Relative mRNA expression of *TBX21* and *RORC* normalized to *EF1α*. Data are mean ± SEM; each dot represents an individual. Analysis of multiple groups was performed by 1-way ANOVA followed by Holm-Šidák’s multiple comparisons test (**B**–**F**). Statistical significance was determined using unpaired (Placebo versus NR) *t* tests (baseline versus visit 2). *P < 0.05, **P < 0.01, ***P < 0.001.

We found that ex vivo NR administration blunts Th1 and Th17 immunomodulation in CD4^+^ T cells isolated from healthy volunteers and participants with psoriasis (13). To validate the in-vivo effect of NR in participants with psoriasis (13), we recruited and obtained consent from 27 volunteers with mild-to-moderate psoriasis to participate in a randomized, oral study comparing NR with a placebo (500 mg twice daily). Participant enrollment is shown on Figure 1A. The study protocol is schematized, with research blood drawn at baseline and after 4 weeks of supplementation (Supplemental Figure 1A; supplemental material available online with this article; https://doi.org/10.1172/jci.insight.203826DS1). The participant demographics showed similar extents of skin involvement in the placebo and NR arms, as measured by the psoriasis area and severity index (PASI) (Supplemental Figure 1B). NR was well tolerated with no serious adverse effects. The participant demographics and adverse events have been reported under the FDAAA 801 rule and published on clinicaltrials.gov under the results posted section.

Following study completion, stored whole-blood NAD^+^ levels were measured, showing elevated NAD^+^ in response to NR versus placebo-supplemented volunteers (Figure 1B). Clinical measurements of the leukocyte differential counts showed no profound effects of NR, although NR modestly and significantly reduced neutrophil levels (Supplemental Table 1). Interestingly, we found this same effect on neutrophil levels in a prior placebo-controlled study in healthy volunteers (Clinical Trial No.: NCT02812238). Data from that study has been published (13), although we did not previously reported on the NR effect on neutrophils from that study. These data are now presented here in Supplemental Table 2. Clinical chemistry measurements revealed no appreciable effects of NR versus placebo on fasting blood glucose, albumin, liver transaminases, or cholesterol levels (Supplemental Table 3). In contrast, direct bilirubin levels were modestly, albeit significantly, higher in the NR-treated participants, although this appears attributable to higher baseline levels. Within-cohort analysis showed that NR reduced blood urea nitrogen (BUN) and total cholesterol levels, whereas no effects were observed in placebo-control participants (Supplemental Table 3). Furthermore, levels of C-reactive protein (CRP), an acute phase reactant that may reflect systemic inflammation, was significantly blunted following NR administration in this study (Supplemental Table 3). As ex vivo NR supplementation has been shown to modulate arginine metabolism (13) and arginine is pivotal for ammonia detoxification in the urea cycle, it should also be noted that NR reduced blood urea nitrate (BUN) levels and the BUN/Creatinine ratio (Supplemental Figure 1, C and D). Consistent with our prior ex vivo study, NR blunted both IFN-γ (IFNg) and IL-17 production in response to T cell receptor (TCR) activation of CD4^+^ T cells (Th0) isolated from psoriatic individuals (Figure 1, C and D), with no effect on the Th2 cytokine IL-4 (Supplemental Figure 1E). These data were confirmed by flow cytometry (antibody panel: Supplemental Table 3). Th1 cells were double-stained with the Th1 transcription factor TBX21 and IFNg (Figure 1E), and Th17 cells were stained with the Th17 transcription factor RORC and IL-17 (Figure 1F). Double staining of Th2 cells with the transcription factor GATA3 and IL-4, and Treg cells with the transcription factor FOXP3 and CD25, similarly showed no changes in response to NR (Supplemental Figure 1, F and G). Parallel analysis of activated Th0 cells showed no change in the expression of *TBX21*, a reduction in *RORC* expression (Figure 1G), and no effect on transcript levels of *GATA3* or *FOXP3* in response to oral NR supplementation (Supplemental Figure 1H).

### RNA-seq analysis identifies the SLIT-ROBO ligand-receptor signaling pathway as responsive to NR Supplementation.

**Figure 2:**
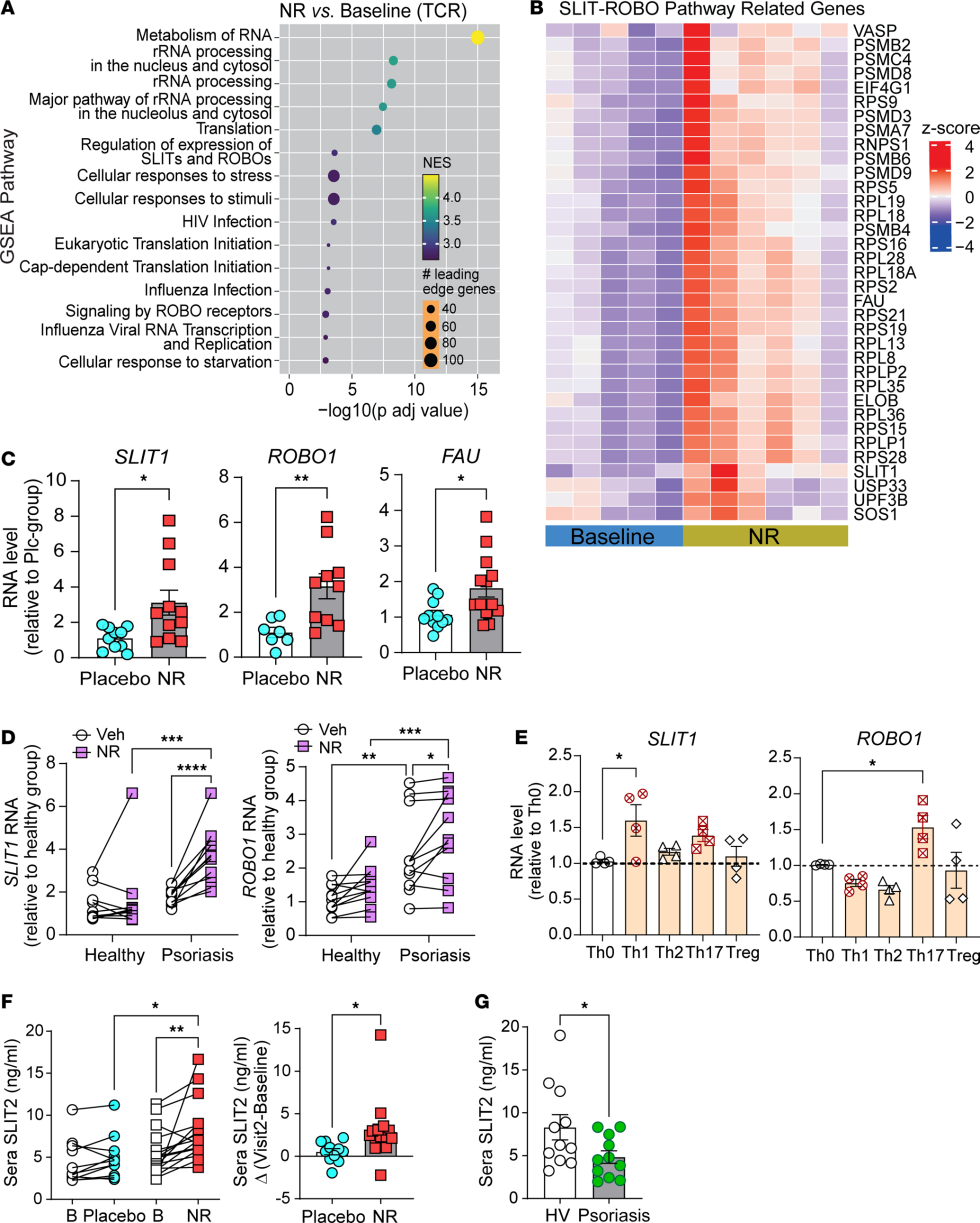
NR induces SLIT–ROBO signaling in psoriatic CD4^+^ T cells. (**A**) Gene Set Enrichment Analysis (GSEA) against C2 Reactome pathways from the Human Molecular Signatures Database (MSigDB). The y axis represents top 15 most significantly enriched pathways with highest NES scores. The x axis represents –log_10_ transformed adjusted *P* values and the dot color is scaled to the Normalized Enrichment Score (NES). (**B**) Heatmap of SLIT–ROBO pathway genes. (**C**) qRT-PCR of *SLIT1*, *ROBO1*, and *FAU* in activated CD4^+^ T cells (*n* = 10–12). Data were normalized to *18S* rRNA. (**D**) Relative mRNA expression of *SLIT1* and *ROBO1* in healthy and psoriatic CD4^+^ T cells in the presence of ex vivo NR and 10% autologous serum (*n* = 11/group, ClinicalTrials.gov Identifiers: NCT01778569 and NCT01143454). (**E**) Relative mRNA expression of *SLIT1* and *ROBO1* in Th1, Th2, Th17, and Treg compared to Th0. (**F** and **G**) Circulating SLIT2 in NR (*n* = 15) versus placebo (*n* = 11), and psoriasis versus healthy controls (*n* = 11/group). Data represent mean ± SEM; each dot indicates an individual participant. Analysis of multiple groups was performed by 2-way ANOVA followed by Holm Šidák’s multiple comparisons test (**D**) and 1-way ANOVA followed by Dunnett (**E**) and Šidák’s multiple comparisons test (**F**). Statistical significance was determined using unpaired 2-tailed Student’s *t* tests (placebo versus NR; HV versus psoriasis). *P < 0.05, **P < 0.01, ***P < 0.001, ****P < 0.0001.

As a discovery approach, bulk RNA-seq was performed on paired CD4^+^ T cells from participants with psoriasis before (baseline) and after 4 weeks of NR supplementation (NR), under both naive and 3-hour TCR-stimulated conditions. Principal component analysis (PCA, PC1 versus PC2) demonstrated separation of status of activation and NR supplementation, with 44.6% of the variance (along PC1) in gene expression data explained by NR supplementation alone, indicating that NR induced global transcriptional changes in CD4^+^ T cells (Supplemental Figure 2A). Differential analyses comparing gene expression in NR versus baseline identified total of 723 (392 up, 331 down) and 491 (129 up, 362 down) significant genes (*P* value < 0.05) in naive and activated T cell states, respectively. Volcano plot highlighted differentially expressed genes (DEGs) pre- versus post-NR in both naive and activated cells (Supplemental Figure 2B and Supplemental Data 1 and 2). Gene Set Enrichment Analysis (GSEA) identified Reactome pathways involved in the regulation of SLITs and ROBOs in both naive and TCR-activated CD4^+^ T cells (Figure 2A, Supplemental Figure 2C, and Supplemental Data 3 and 4). Heatmaps show that the genes associated with SLIT-ROBO pathway were uniformly upregulated in response to NR in both naive and TCR-activated cells (Figure 2B and Supplemental Figure 2D). To validate these transcript-level changes, we quantified the expression of SLIT and ROBO genes in CD4^+^ T cells. The levels of *SLIT2*, *SLIT3*, *SLIT4,* *ROBO2*, *ROBO3*, and *ROBO4* were either not detectable or expressed at very low abundance (data not shown). In contrast, the genes encoding SLIT1 and ROBO1 were expressed in both naive and TCR-activated CD4^+^ T cells. In naive cells, NR supplementation increased *ROBO1* but not *SLIT1* transcript levels (Supplemental Figure 2E). In TCR-activated cells, both *SLIT1* and *ROBO1* transcripts were induced by in vivo NR supplementation (Figure 2C). To further validate the RNA-seq data, we assayed the transcript levels of the ubiquitin-like and ribosomal protein S30 fusion protein (FAU), which was also significantly induced in both naive and activated CD4^+^ T cells (Figure 2C and Supplemental Figure 2E). To evaluate the effect of psoriasis, we analyzed cDNA from our prior ex vivo NR administration study in primary CD4^+^ T cells from healthy control and psoriasis participants (13). In activated cells, NR significantly increased *SLIT1, ROBO1,* and *FAU* transcript levels in psoriasis but not in healthy control CD4^+^ T cells (Figure 2D and Supplemental Figure 2F). Interestingly, *ROBO1* expression was elevated at baseline and further augmented by NR in the psoriasis cohort (Figure 2D). We then assayed the transcript levels of *SLIT1* and *ROBO1* during primary CD4^+^ T cell polarization. Transcript levels of *SLIT1* were significantly upregulated following Th1 polarization, and RNA and protein levels of ROBO1 were induced by Th17 polarization (Figure 2E and Supplemental Figure 2G). To extend these findings, we measured circulating levels of the SLIT isoforms. SLIT1 was undetectable in this cohort of psoriasis participants (data not shown), SLIT2 was measurable in the low ng/ml range, and SLIT3 was detectable at less than 1 ng/ml (Figure 2F and Supplemental Figure 2H). NR supplementation significantly increased circulating SLIT2 levels (Figure 2F) without affecting SLIT3 levels in serum from the in vivo intervention study (Supplemental Figure 2H). Circulating SLIT2 was reduced in psoriasis participants relative to healthy controls, whereas SLIT3 levels were similar between groups (Figure 2G and Supplemental Figure 2I). The bioinformatics data clearly uncovered the regulation of the SLIT-ROBO pathway in response to NR. At the same time, the quantification of transcript levels, which may not directly reflect biological activity, showed variable results. However, circulating levels of SLIT2, as the ligand in this pathway, were both altered in psoriasis and modified by NR. Hence, given the reduced levels of circulating SLIT2 in psoriasis, its induction by NR and the robust expression of its cognate receptor ROBO1 in CD4^+^ T cells, we focused further analysis on this pathway.

### SLIT2 mimics the effect of NR on dampening Th17 immune responsiveness.

**Figure 3:**
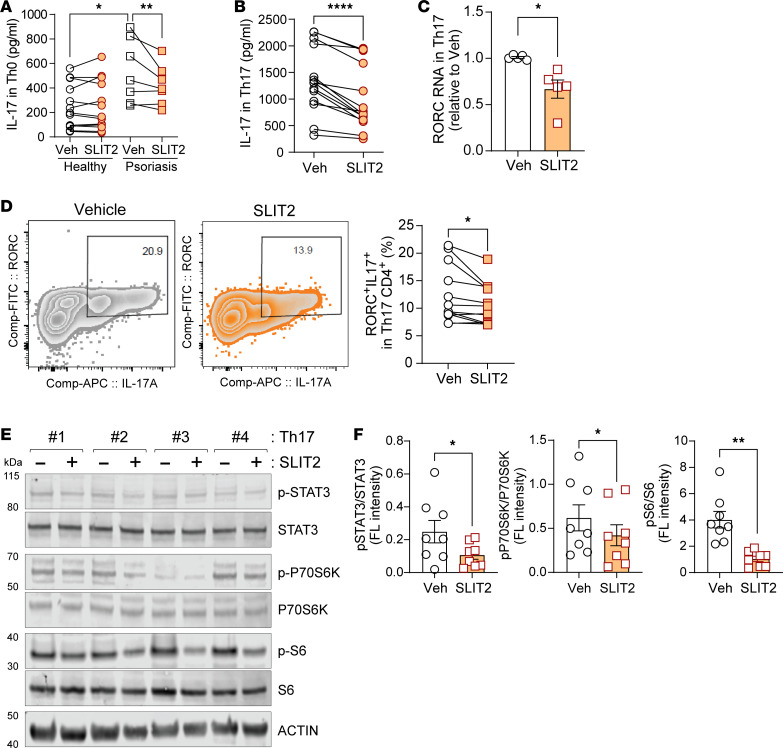
SLIT2 mimics NR effects on Th17 immune responses. (**A**) IL-17 secretion from psoriatic Th0 cells treated with recombinant SLIT2 (200 ng/ml) for 72 hrs (*n* = 13 for healthy volunteers and *n* = 7 for psoriatic participants). (**B**) IL-17 secretion in Th17-polarized CD4^+^ T cells with SLIT2 (*n* = 14). (**C**) Relative mRNA expression of *RORC* in SLIT2-treated Th17 cells. Data were normalized to *EF1a* reference gene (*n* = 5). (**D**) Flow cytometry of RORC^+^IL-17^+^ from pathogenic Th17 cells treated with SLIT2 treatment (*n* = 12). (**E** and **F**) Immunoblot of p-STAT3, p-P70S6K, and p-S6 in Th17 cells treated with vehicle or SLIT2 (*n* = 7–8). The immunoblots in this figure are cropped images and the full membranes are depicted in an additional supplemental file. The data point of each participant were shown as dots and all data were represented as mean ± SEM. Analysis of multiple groups was performed by 1-way ANOVA followed by Šidák’s multiple comparisons test (**A**). The *P* values for comparisons of 2 groups were calculated using paired 2-tailed Student’s *t* test (Veh versus SLIT2). Veh, vehicle. *P < 0.05, **P < 0.01, ****P < 0.0001.

Given our prior data showing that NR preferentially suppresses Th17 activation (13) and that *ROBO1* expression was highest in Th17 cells and induced by NR, we next assayed the effect of endogenous SLIT2 on primary CD4^+^ T cells. As previously observed, IL-17 levels were elevated in Th0 cells from participants with psoriasis compared with participants in the healthy control group. Incubation with recombinant SLIT2 significantly blunted IL-17 secretion in the psoriasis cohort (Figure 3A). This inhibitory effect of SLIT2 on IL-17 release was dose dependent (Supplemental Figure 3A) and also evident in primary CD4^+^ T cells polarized toward the Th17 lineage (Figure 3B). In parallel, SLIT2 diminished the expression of the Th17 polarizing transcription factor *RORC* (Figure 3C). Flow cytometry confirmed these findings, demonstrating that SLIT2 incubation decreased the frequency of RORC^+^IL-17^+^ psoriatic Th17 cells (Figure 3D and Supplemental Figure 3B). To distinguish between nonpathogenic and pathogenic Th17 subsets, CD4^+^ T cells were differentially polarized. SLIT2 preferentially blunted IL-17 secretion from pathogenic Th17 cells (Supplemental Figure 3, C and D). Consistent with these transcriptional and cytokine findings, SLIT2 also attenuated canonical Th17 signaling, evidenced by reduced phosphorylation of STAT3 and downstream mTOR pathway regulators P70S6k, and S6, in Th17 cells (Figure 3, E and F). Finally, in transwell migration assays, SLIT2 significantly reduced activated Th17 cell migration (Supplemental Figure 3E).

### ROBO1 signaling is required for SLIT2-mediated suppression of Th17 activation.

**Figure 4:**
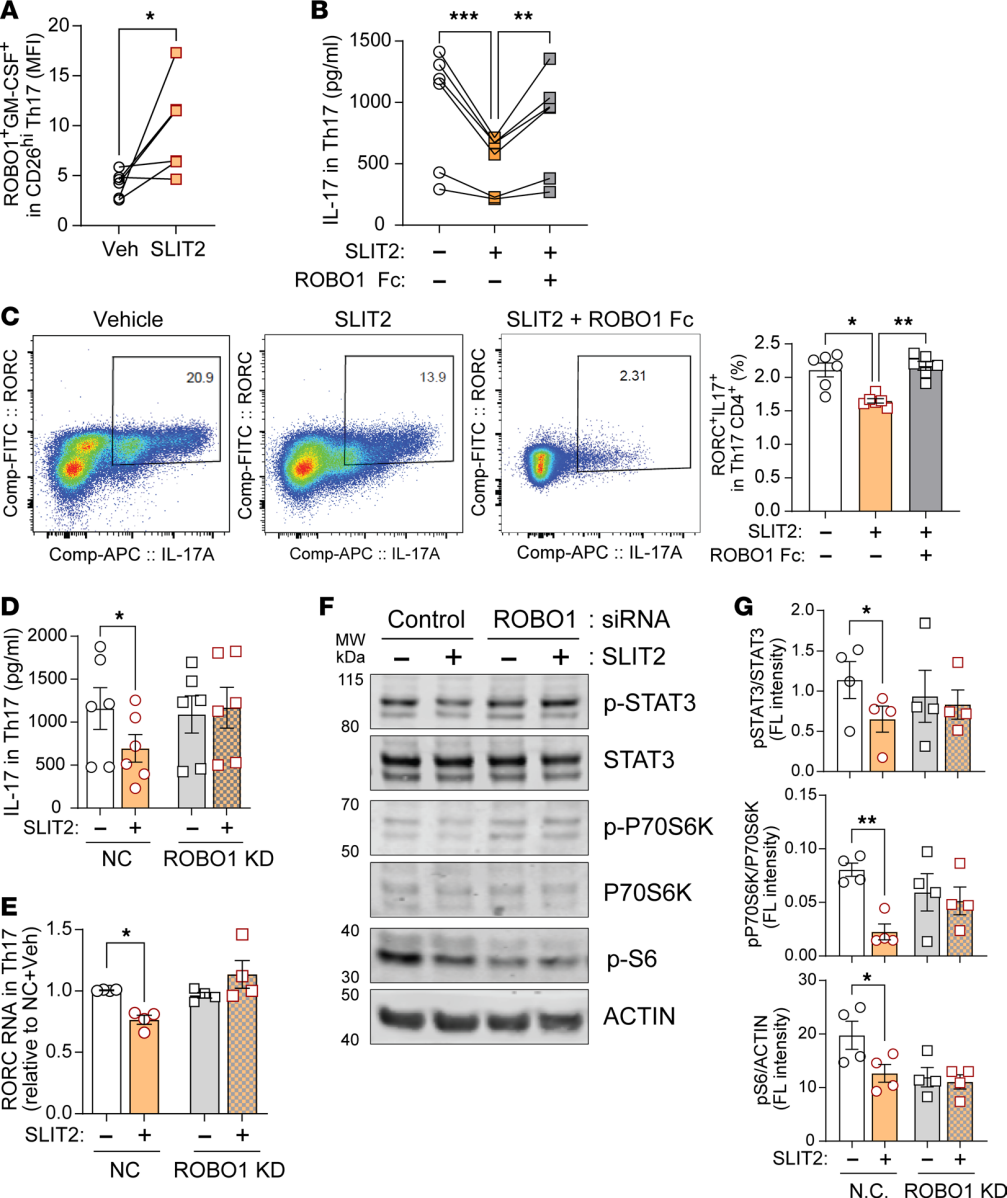
ROBO1 specificity is required for SLIT2-mediated immunomodulation in psoriatic Th17 cells. (**A**) Surface expression of ROBO1 in Th17 cells treated with SLIT2 (*n* = 6). (**B** and **C**) IL-17 secretion (ELISA) and Th17 population (RORC^+^IL-17^+^, flow cytometry) in Th17 cells treated with recombinant ROBO1 Fc fusion protein (600 μg/mL) for 72 h. (**D**) IL-17 secretion in ROBO1 knockdown (KD) Th17 cells with vehicle or SLIT2 treatment (*n* = 8). (**E**) *RORC* mRNA expression in control or ROBO1 KD Th17 cells (*n* = 4). (**F** and **G**) Immunoblot quantification of p-STAT3, p-P70S6K, and p-S6 in ROBO1 KD Th17 cells treated with vehicle or SLIT2 (*n* = 4). Full-length immunoblots are provided in an additional supplemental file. Each data point represents an individual participant; bars show mean ± SEM. Statistical significance was determined by 1-way ANOVA followed by Šidák’s multiple comparisons test and paired 2-tailed Student’s *t* test (Veh versus SLIT2). Veh, vehicle. *P < 0.05, **P < 0.01, ***P < 0.001.

We measured the surface expression of ROBO1 in pathogenic Th17 cells treated with SLIT2. Flow cytometry showed that ROBO1 expression was increased by SLIT2 in these cells (Figure 4A). To determine whether the ROBO1 receptor is required for SLIT2-mediated immunomodulation, a recombinant soluble ROBO1-Fc fusion protein functioning as a ligand trap and competitive inhibitor of ROBO1 signaling was coincubated with SLIT2 in Th17 cells. The soluble ROBO1-Fc impaired the ability of SLIT2 to suppress IL-17 secretion in activated Th17 cells (Figure 4B). Flow cytometry further confirmed that blockade of SLIT2 signaling by soluble ROBO1-Fc reversed the SLIT2-induced reduction of RORC^+^IL-17^+^ cell frequency in psoriatic Th17 cells (Figure 4C). ROBO1 was then transiently depleted in Th17 cells using siRNA, resulting in approximately 60% reduction in *ROBO1* transcript levels (Supplemental Figure 4A). ROBO1 knockdown abrogated the suppressive effects of SLIT2 on both IL-17 secretion and the frequency of RORC^+^IL-17^+^ Th17 cells (Figure 4D) and prevented SLIT2 from downregulating *RORC* expression (Figure 4E). In parallel, ROBO1 depletion abolished the inhibitory effects of SLIT2 on STAT3, P70S6K, and S6 phosphorylation, restoring canonical Th17 signaling activation (Figure 4, F and G). In contrast, siRNA-targeted knockdown (KD) of *SLIT1* in Th17 cells did not alter the capacity of exogenous SLIT2 to suppress *RORC* expression or IL-17 secretion (Supplemental Figure 4, B–D), confirming the specificity of SLIT2-ROBO1 signaling in mediating these effects.

### RhoGTPase signaling mediates SLIT2-dependent immunomodulation.

**Figure 5:**
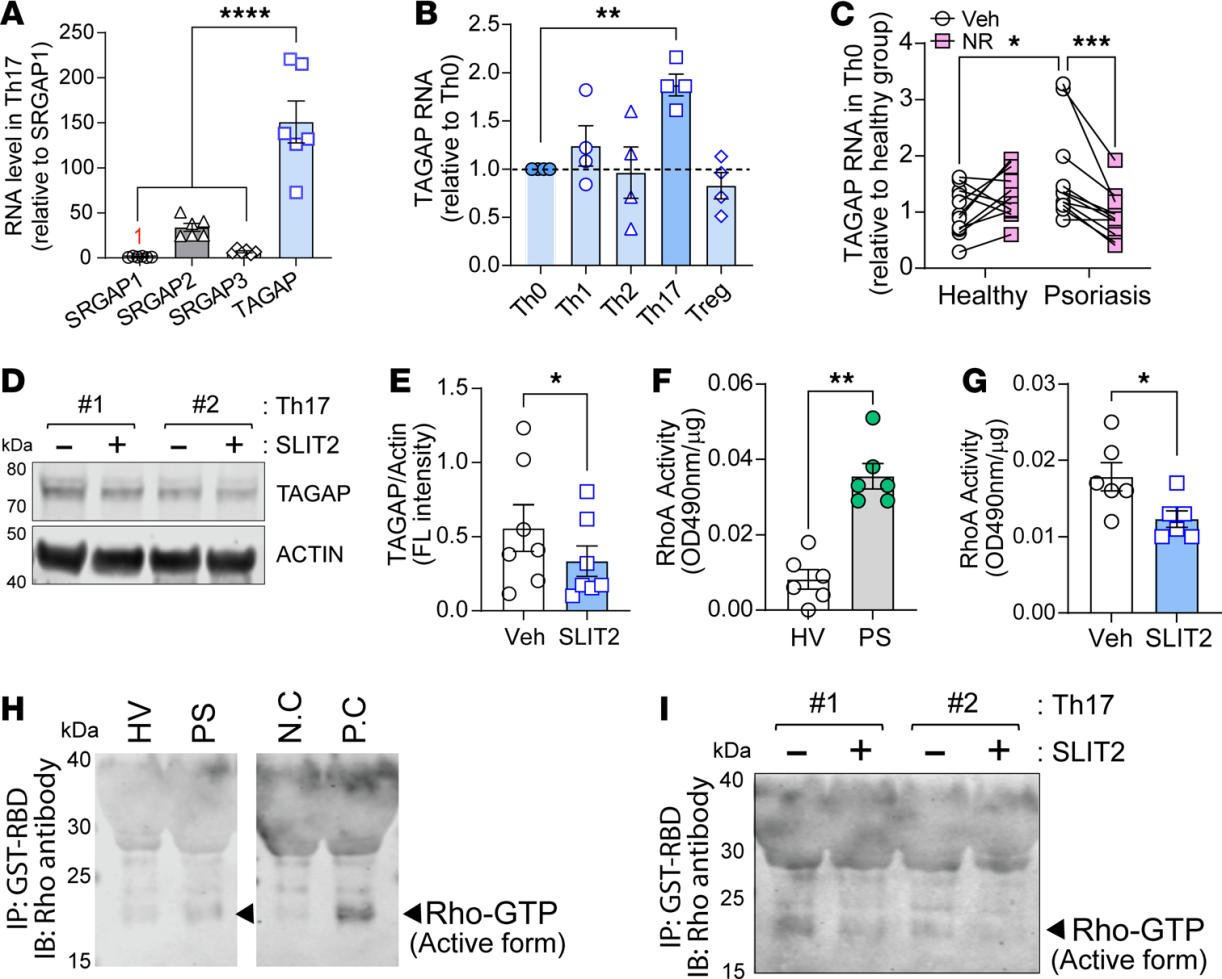
In vivo NR blunts Rho GTPase activity in naive and activated psoriatic CD4^+^ T cells. (**A**) Relative transcript levels of SLIT-ROBO–associated Rho GTPase–activating proteins (*SRGAP1-3*) and *TAGAP* in psoriatic Th17 cells, normalized to *SRGAP1*. (**B**) *TAGAP* transcript levels in differentiated Th subsets (Th1, Th2, Th17, and Treg) compared with nondifferentiated CD4^+^ T cells (Th0). (**C**) *TAGAP* mRNA in CD4^+^ T cells from healthy and psoriatic participants in the presence of ex vivo NR and 10% autologous serum (*n* = 11/group). (**D** and **E**) Representative immunoblot and quantification of TAGAP in pathogenic Th17 cells treated with vehicle or SLIT2 (*n* = 7). Protein abundance was normalized to actin. (**F** and **G**) Rho GTP-binding activity in healthy versus psoriatic CD4^+^ T cells and vehicle or SLIT2-treated Th17 cells (*n* = 6 per group). (**H** and **I**) Active Rho pull-down assay using GST-Rhotekin-binding domain (RBD) and immunoblot for Rho in healthy versus psoriatic CD4^+^ T cells (**H**) and vehicle versus SLIT2-treated Th17 cells (**I**). GDP- and GTPγS-treated lysates were used as negative (N.C.) and positive (P.C.) controls. All qPCR data were normalized to *18S* rRNA or *β-ACTIN*. Each point represents an individual participant; bars show mean ± SEM. Statistical significance was determined by unpaired (placebo versus NR) or paired (Veh versus NR or Veh versus SLIT2) 2-tailed Student’s *t* test or 1-way ANOVA with Šidák’s multiple comparisons test. *P < 0.05, **P < 0.01, ***P < 0.001, ****P < 0.0001. Veh, vehicle.

A canonical signaling pathway downstream of SLIT-ROBO signaling includes the intracellular domain of ROBO interacting with Rho GTPase activating proteins (GAPs) (18, 19). We therefore examined the expression of more ubiquitously expressed SLIT-ROBO GAPs (SRGAPs) and the T cell-enriched Rho GTPase, TAGAP (20) in Th17-polarized CD4^+^ T cells. Among these, the expression of *TAGAP* was markedly higher than *SRGAP* isoforms, with *SRGAP2* being the most abundant *SRGAP* in Th17 cells (Figure 5A). During CD4^+^ T cell polarization, *TAGAP* expression was detected across all T helper lineages, but was most enriched in Th17 cells (Figure 5B). Both TAGAP and SRGAPs regulate the small Rho GTPase family (RhoA, Rac, and CDC42) as GTPase activating proteins. Consistent with this regulation, NR reduced *TAGAP* and *SRGAP2* transcript levels in both naive and Th0 cells relative to the placebo control group (Supplemental Figure 5, A and B). To test whether NR effects were preserved ex vivo, we quantified *TAGAP* and *SRGAP2* transcript levels in TCR-activated CD4^+^ T cells from individuals who were healthy and had psoriasis. Interestingly, transcript levels of *TAGAP* were elevated at baseline in psoriasis and exclusively blunted by NR treatment in psoriatic cells (Figure 5C and Supplemental Figure 5C). In Th17-polarized CD4^+^ T cells from participants with psoriasis, SLIT2 treatment reduced steady-state TAGAP protein levels (Figure 5, D and E).

GTPase activity assays showed that Rho GTPase activity was elevated in psoriatic CD4^+^ T cells relative to healthy volunteers (Figure 5, F and H), and SLIT2 attenuated RhoA GTPase activity and GTP-binding affinity in psoriatic Th17 cells (Figure 5, G and I). In contrast, the GSEA of TCR-activated cells from the NR intervention did not identify GTPase-related pathways, but we showed strong evidence of reactive oxygen species–associated (ROS-associated) signatures. This regulation following in vivo NR supplementation parallels the prior findings of reactive oxygen species regulatory effects in response to ex vivo NR supplementation in CD4^+^ T cells (Supplemental Figure 5D) (13). To evaluate whether SLIT2 recapitulates this metabolic regulation, SLIT2-treated Th17 cells displayed reduced total cellular and mitochondrial ROS, as measured by DCFDA and MitoSox assays, respectively (Supplemental Figure 5, E and F).

### RhoGTPase signaling is regulated through the interaction between ROBO1 and TAGAP.

**Figure 6:**
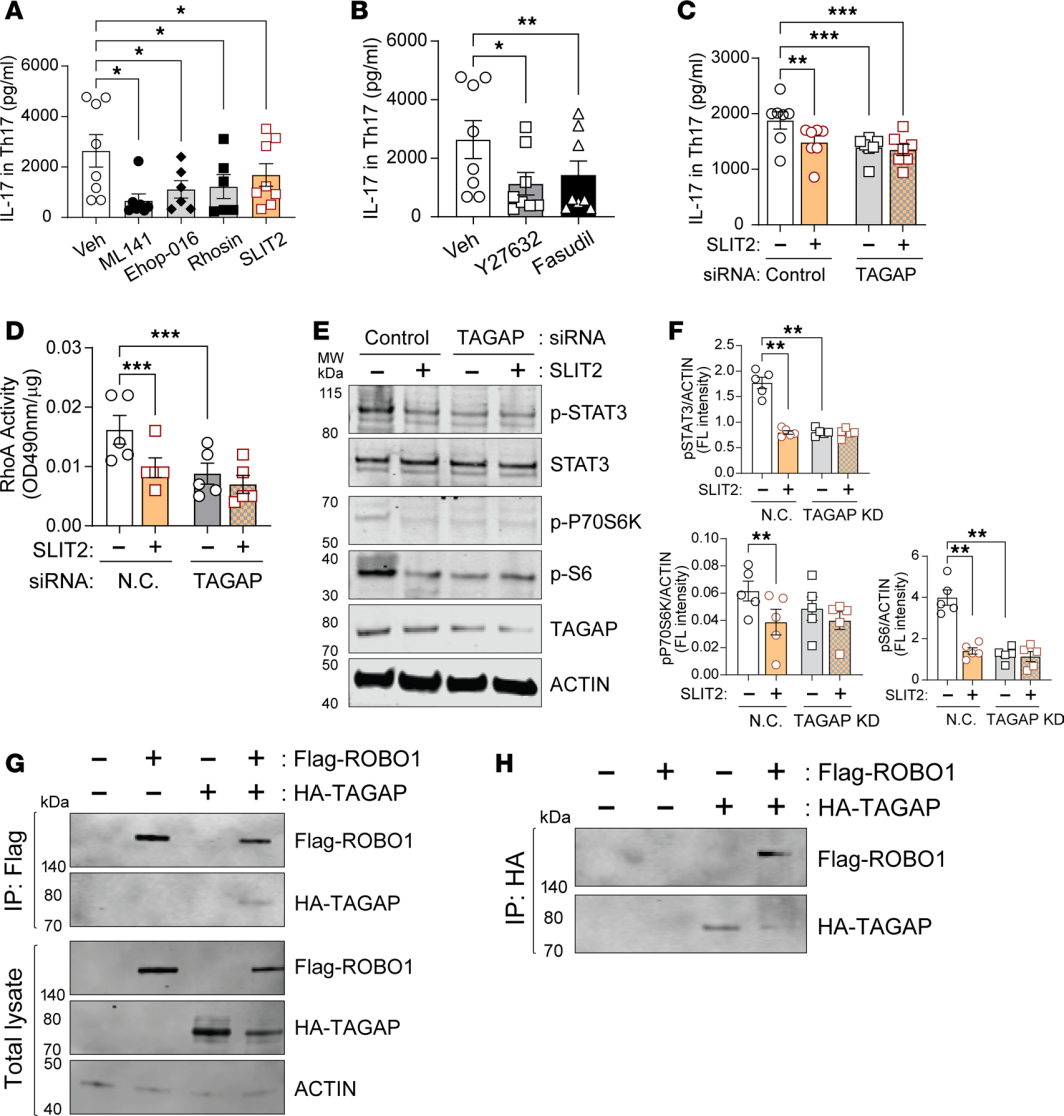
TAGAP mediates SLIT2 regulatory effects in psoriatic Th17 cells. (**A** and **B**) IL-17 secretion from Th17 cells treated with Rho GTPase inhibitors (20 μM ML141 for CDC42; 10 μM Ehop-016 for Rac; 30 μM Rhosin for Rho) or ROCK inhibitors (10 μM Y27632 for ROCK1/2; 10 μM Fasudil for ROCK2) for 24 hrs (*n* = 8). (**C**) IL-17 secretion from TAGAP KD Th17 cells treated with vehicle or SLIT2 (*n* = 7). (**D**) Rho GTP-binding activity in control or TAGAP KD Th17 cells (*n* = 5). Rho activity was normalized to total protein amount. (**E** and **F**) Immunoblot quantification of p-STAT3, p-P70S6K, and p-S6 in TAGAP KD Th17 cells treated with vehicle or SLIT2 (*n* = 5). (**G** and **H**) Coimmunoprecipitation (IP) demonstrating physical interaction between TAGAP and ROBO1 in 293 cells transfected with 3 × HA-TAGAP and 3 × Flag-ROBO1. IP was performed using anti-Flag M2 magnetic beads and blotted with anti-HA antibody; total lysates shown below (**G**). Reciprocal IP using anti-HA magnetic beads followed by IB with anti-ROBO1 antibody (**H**). All immunoblots are cropped; full-length membranes are shown in an additional supplemental files. Data points represent individual donors and are shown as dots. Data are presented as mean ± SEM and analyzed by 1-way ANOVA followed by Šidák’s multiple comparisons test. *P < 0.05, **P < 0.01, ***P < 0.001.

To determine whether SLIT2 blunts Th17 activity by inhibiting GTPase signaling, selective inhibitors targeting individual GTPases were compared with recombinant SLIT2 treatment. Pharmacologic inhibition of CDC42 (ML141), RAC (EHop-016), or RHO (Rhosin) each suppressed IL-17 secretion from psoriatic Th17-polarized cells to a similar extent as SLIT2 (Figure 6A). This blunting effect on IL-17 secretion was also evident after inhibition of the downstream Rho-associated kinase (ROCK) using Y27632 or Fasudil (Figure 6B).

This regulatory effect was dependent on TAGAP, as siRNA-targeted *TAGAP* KD attenuated SLIT2’s ability to blunt IL-17 release, although TAGAP KD alone also reduced IL-17 secretion (Figure 6C and Supplemental Figure 6A). TAGAP KD had a similar effect to SLIT2 on blunting RhoA GTPase activity and phosphorylation of STAT3 and S6 (Figure 6, D–F), and similarly reduced *RORC* transcript levels, which were not further blunted by SLIT2 treatment (Supplemental Figure 6B). Coimmunoprecipitation assays revealed a physical interaction between TAGAP and the ROBO1 receptor, supporting a direct signaling link between SLIT2-ROBO1 and Rho GTPase regulation by TAGAP (Figure 6, G and H). In contrast, the siRNA KD of SRGAP2 did not attenuate the ability of recombinant SLIT2 to dampen Th17 polarization or activation, reinforcing the predominant role of the TAGAP GTPase in SLIT2-mediated immunomodulation in Th17 cells (Supplemental Figure 6, C–E).

### Dermal fibroblasts are a source of SLIT2 production in response to NR administration.

**Figure 7:**
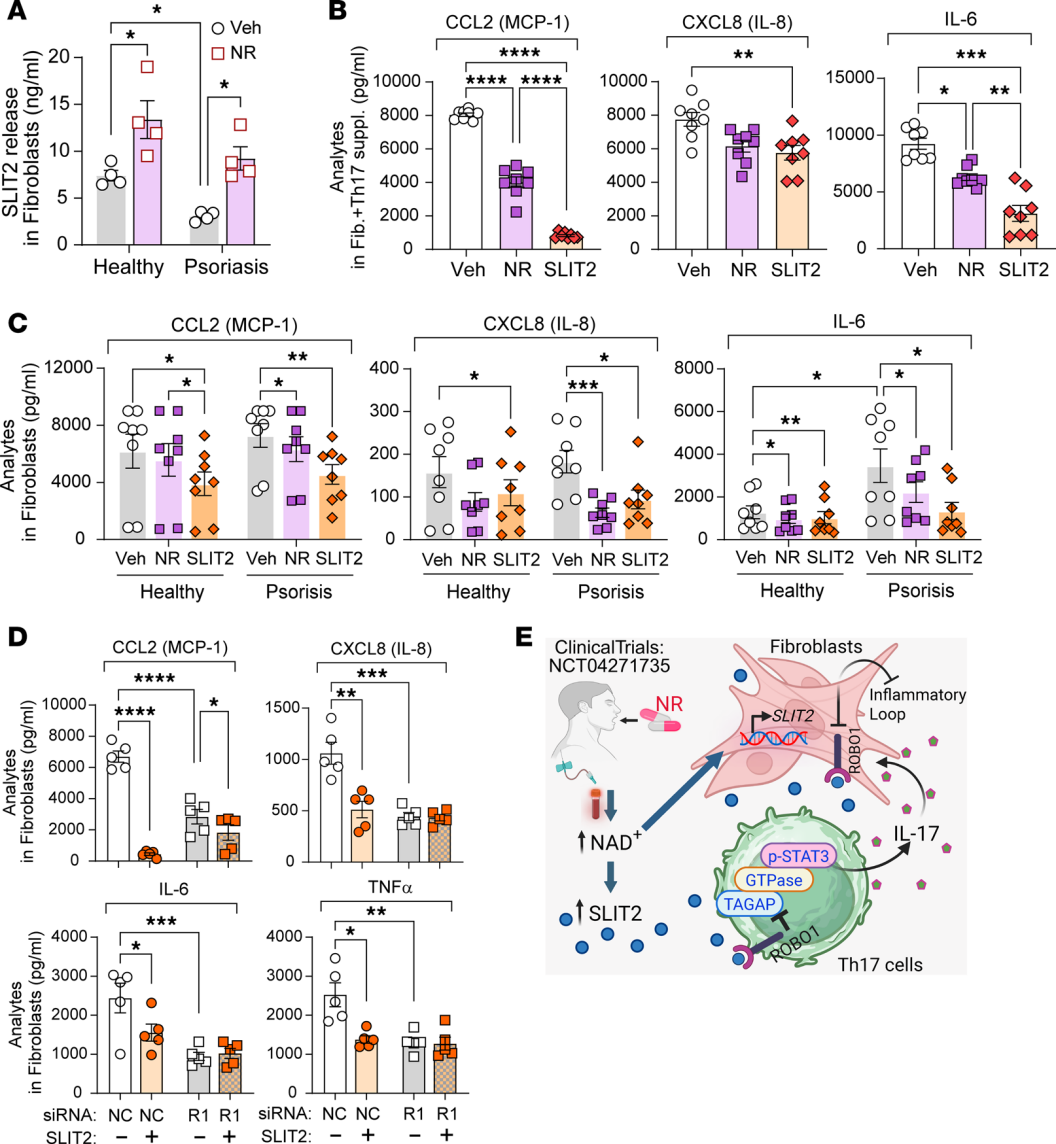
NR reduces fibroblast inflammatory responses via SLIT2 signaling. (**A**) SLIT2 production in skin fibroblasts from participants who were healthy and had psoriasis (*n* = 4 per group) after NR supplementation. Biopsies collected by punch under local anesthesia (ClinicalTrials.gov Identifiers: NCT01778569 and NCT01143454). Lesional skin used for participants with psoriasis. (**B–D**) CCL2, CXCL8, and IL-6 secretion measured by 13-plex bead-based multiplex assay. (**B**) Healthy fibroblasts incubated with Th17 differentiation media (*n* = 8 replicates, 4 independent experiments). (**C**) Fibroblasts from healthy (HV) and psoriasis (PS) participants. (**D**) SLIT2 effects on fibroblasts transfected with control or ROBO1 siRNA (*n* = 5). (**E**) Proposed model of NR-mediated immunoregulation. NR increases NAD^+^ levels and enhances SLIT2 secretion from fibroblasts and other cell types, leading to ROBO1-dependent suppression of the Th17 response. Data: mean ± SEM; statistical analyses: unpaired 2-tailed *t* test (HV versus PS), paired *t* test (Veh versus NR), or 1-way ANOVA for multiple-group comparisons. *P* < 0.05, *P < 0.01, **P < 0.001, ***P < 0.0001. Veh, vehicle.

Psoriatic inflammation arises in par from dysregulated signaling among dermal innate, adaptive, and nonprofessional immune cells (21). To investigate whether NR modifies this inflammatory crosstalk, skin punch biopsies were obtained from healthy volunteers and from the lesional skin of participants with psoriasis (Supplemental Figure 7A). Primary dermal fibroblasts were cultured for biochemical analysis. Quantitative PCR analysis of SLIT and ROBO family transcripts revealed that *SLIT2* and *SLIT3* were markedly enriched compared with *SLIT1* in primary fibroblasts, whereas their expression was diminished in psoriasis-derived fibroblasts compared with controls (Supplemental Figure 7B). The transcript level of *SLIT4* and *ROBO3* were undetectable (data not shown). Among ROBO isoforms, *ROBO1* was most abundant, and expression of *ROBO1*, *ROBO2*, and *ROBO4* transcripts were elevated in psoriasis fibroblasts relative to healthy controls (Supplemental Figure 7, C and D). Importantly, this transcriptional increase in *ROBO1* was also confirmed at the protein level by flow cytometric analysis (Supplemental Figure 7D). Ex vivo NR supplementation increased transcript levels of *SLIT2* and enhanced SLIT2 protein release from both healthy and psoriatic fibroblasts, without altering *ROBO1* expression (Figure 7A and Supplemental Figure 7E). When primary human fibroblasts were exposed to conditioned media with Th17-differentiation supplements, secretion of IL-6 and TNFa was elevated but was significantly reduced by the concurrent exposure to recombinant SLIT2 (Supplemental Figure 7F). Comparative assays demonstrated that SLIT2 exerted a stronger antiinflammatory effect than NR supplementation, in more effectively reducing the canonical fibroblast chemokines, CCL2 and CXCL8 and cytokine, IL-6 (Figure 7B). This enhanced SLIT2-mediated immunomodulation was operational in both healthy and psoriatic fibroblasts. Although baseline IL-6 secretion was higher in psoriatic fibroblasts, both NR and SLIT2 significantly reduced its levels (Figure 7C). Finally, siRNA-mediated *ROBO1* KD specifically disrupted the antiinflammatory effects of SLIT2 in healthy fibroblasts, confirming that the SLIT2-ROBO1 axis mediates this regulatory response (Figure 7D and Supplemental Figure 7G). Together, these findings support NR-mediated immunoregulation in psoriasis, whereby NR-induced NAD^+^ elevation enhances SLIT2 levels, which act in a paracrine manner on CD4^+^ T cells via ROBO1 to suppress Th17 responsiveness via modulation of TAGAP/RhoA/STAT3 signaling (Figure 7E).

## Discussion

The major findings from this study are that increasing whole-blood NAD^+^ levels in response to in-vivo NR supplementation dampened CD4^+^ T cell, Th1, and Th17 immune responsiveness without affecting Th2 or Treg regulatory programs. Moveover, these immune-regulatory effects in Th17 cells were mediated, in part, via activation of the SLIT2-ROBO1 signaling pathway through the inhibition of T cell–enriched Rho GTPase-TAGAP. NR supplementation further increased circulating SLIT2 levels, and in primary skin fibroblasts, NR increased SLIT2 production while concurrently blunting fibroblast inflammatory activity. Interestingly, IL-6 levels were elevated in fibroblasts extracted from psoriatic participants compared with controls, and NR similarly dampened IL-6 production in these nonprofessional immune cells.

Previously we had shown that NR preferentially reduced Th17 immune responsiveness in CD4^+^ T cells extracted from people with psoriasis. This study was designed as a short-term, placebo-controlled study to evaluate biological effects rather than clinical efficacy of oral NR supplementation in participants with psoriasis. Nevertheless, and consistent with NR oral supplementation in other diseases, including heart failure, Parkinson’s disease, and insulin resistance, supplementation with this vitamin B3 analogue was well tolerated. Although not fully explored, the most interesting potential leukocyte effect was that NR reduced absolute and relative neutrophil levels. Given the importance of NETosis in inflammatory and autoimmune diseases such as psoriasis and SLE, this regulation warrants further investigation.

The SLIT proteins are secreted glycoproteins initially discovered as extracellular ligands that guide cell migration during neuronal organogenesis. Their recognized biological roles have since expanded to include regulation of organ development, angiogenesis, inflammatory cell chemotaxis, and tumor cell migration and metastasis (22). SLIT1 is predominantly synthesized in the brain, whereas SLIT2 and SLIT3 are more ubiquitously expressed. The ROBO1 receptor is similarly broadly expressed, as is SRGAP1. In contrast, the TAGAP transcript is predominantly enriched in the bone marrow, although the protein itself is detected across multiple tissue types. Notably, single-cell RNA-seq data show that *TAGAP* is most highly expressed in neutrophils, but also present across other leukocyte lineages (23).

In this study, we find that participants with psoriasis have lower circulating SLIT2 levels compared to healthy controls and that SLIT2 levels are induced by NR. Although the tissue source of circulating SLIT2 has not been directly identified, we demonstrate that NR supplementation increases SLIT2 secretion from primary skin fibroblasts from both healthy volunteers and individuals with psoriasis. ROBO1 expression is also higher in CD4^+^ T cells extracted from participants with psoriasis and is highest within the Th17 lineage. TAGAP transcript levels are also enriched in CD4^+^ T cells in psoriasis, enriched in the Th17 lineage, and attenuated in response to NR supplementation. Furthermore, loss-of-function studies identify that SLIT2-mediated inhibition of Rho-GTPase activity via ROBO1 signaling contributes to downstream of NR-dependent suppression of Th17 immune responsiveness. Supporting these findings, SNP analyses have linked TAGAP to the pathogenesis of multiple inflammatory and autoimmune diseases, including psoriasis (24). Knockout mouse studies similarly demonstrated that TAGAP is required for Th17 cell differentiation and that TAGAP depletion ameliorates the pathological features of experimental autoimmune encephalomyelitis and rheumatoid arthritis (25, 26). At a mechanistic level, this biology supports a model in which TAGAP interacts with the TCR to attenuate receptor activity below the Th17-cell differentiation threshold (25). Interestingly, broader GTPase biology may play a role in Th17 biology as the Rac family small GTPase 1 (Rac1) functions as a component of that Tiam1/Rac1 complex binding to, and transactivating, the Th17 transcription factor RORgt (27).

A broader role of SLIT2 in immunoregulation has also begun to be emerge. SLIT2 has been shown to exhibit antitumor effects *via* macrophage M1 polarization accompanied by increased glycolysis and reduced fatty acid oxidation rate (28). Furthermore, macrophage SLIT2/ROBO1 signaling suppresses mTORC1 to augment lysosomal biogenesis and phagocytic bacterial killing (29). mTORC1 has been shown to be essential for murine Th17 cell differentiation (30), and our data similarly show that this pathway is blunted by SLIT2 in human Th17 cells. Additionally, in the isolated perfused heart, SLIT2 overexpression blunted the cardiac inflammatory response by reducing NFkB and NLRP3-linked cytokine secretion (31). The only previous data directly penitent to T cells, as described earlier, relate to Jurkat cell chemotaxis (16). In this study, we demonstrate that augmenting NAD^+^ levels increases circulating SLIT2 and that recombinant SLIT2 attenuates Th17 cell responsiveness via the canonical SLIT/ROBO signaling pathway and via the dampening of mTOR signaling and blunts Th17 cell migration.

Because ROBO receptors are transmembrane proteins, they may be amenable at therapeutic targets. In this vein, the SLIT/ROBO pathway is already being evaluated as a potential therapeutic axis in a range of cancers (32). The present findings, together with prior observations, raise the possibility that agonism of the SLIT/ROBO pathway may ameliorate inflammation. Additionally, given that NR enhances SLIT2 production in skin fibroblasts, the concept that topical niacinamide (33) or a SLIT2 agonist could be developed for dermatologic psoriasis warrants exploration. It would also be informative to determine whether topical niacinamide could upregulate SLIT2 in other inflammatory skin conditions.

The major limitation of this study includes its short duration and relatively small number of study participants. Given the number of human participants with different diseases where NR supplementation is being explored, it is probably appropriate to develop a larger efficacy-powered study to explore the role of NR in inflammatory diseases such as psoriasis (3). In that context, mechanistic studies could also be directly assessed as to the effect of NR on psoriatic skin lesions. Moreover, mechanistic studies need to be pursued to explore the direct link between increasing circulating NAD^+^ levels with increasing SLIT2 secretion and to evaluate the organ system that releases this glycoprotein ligand into circulation. Finally, the data showing that circulating CRP and neutrophil levels are blunted by oral NR supplementation warrant more in-depth analysis of the broader antiinflammatory effects of NR in human disease. We expect to contribute to this knowledge following completion and analysis of our placebo-controlled NR supplementation study in systemic lupus erythematosus (NCT06032923). Furthermore, an ongoing open-label extension study of NR in Parkinson’s disease (NCT05546567) will also explore the antiinflammatory properties of this supplement. Lastly, whether NR can be employed as a steroid- or immunomodulatory-sparing agent is another concept that warrants further investigation.

In conclusion, this study further advances our understanding of how increasing circulatory NAD^+^ levels through oral nicotinamide riboside supplementation exerts direct immunomodulatory effects in a systemic inflammatory disease, psoriasis. Moreover, this study identifies a novel NAD^+^-responsive SLIT2/ROBO1/TAGAP, i.e., ligand-receptor-GTPase signaling pathway that dampens Th17 immune cell responsiveness. Whether this pathway can be manipulated in vivo to attenuate Th17-linked pathology awaits further exploration.

## Methods

### Sex as a biological variable.

Our in vitro supplement study examined men and women participants and the data was analyzed as a combined cohort. The samples sizes were designed to study mechanisms, rather than efficacy and the sample size was too small to separate analyses by sex. To explore the ex vivo effects of NR supplementation skin biopsy samples were studied from male and female participants and male participants were consented for the primary CD4+ T cell experiments.

### Study design and participants.

The Placebo and NR pilot study was registered in ClinicalTrials.gov with the registration number NCT04271735, approved by NHLBI IRB, and performed at the National Institutes of Health (NIH) Clinical center. Study subjects were screened prior to signing consent and subjects were then randomly assigned at a 2:1 ratio to 4-week supplementation with either NR (500 mg bid) or matching placebo (Figure 1A). Randomization was performed by the NIH Clinical Center pharmacy using a block design and investigators, staff and participants were blinded to randomization until enrollment completed and data interpretation required segmentation. The study protocol and a schematic and characteristics are described in Supplemental Figures 1, A and B. The blood from healthy volunteers for functional study were obtained from subjects that consented to enroll on the Disease discovery protocol (NCT01143454) and from the NIH Clinical center blood bank (NCT00001846). In vitro studies to explore mechanism was performed in male mild to moderate psoriasis participants and age-matched control subjects recruited at NIH Clinical Center and provided consent on the NHLBI IRB approved protocols (ClinicalTrials.gov registration numbers NCT01934660 and NCT01143454) respectively (3). The skin biopsy were obtained from the these latter two protocols following informed consent.

### LC-MS measurement of NAD^+^ in whole blood.

Snap-frozen whole blood sample (~100 μl) was thawed at 30°C for 2 mins in a heat block. From the thawed sample, 90 μl of blood was added to a 1.5 ml tube containing 1 μg of internal standard BMP (8-Bromoadenosine 5’-monophosphate). Immediately, 300 μl of 4% cold trichloroacetic acid (TCA) was added and incubated on ice for approximately 20 min to precipitate proteins from the sample. Followed by that, samples were centrifuged at approximately 17,000 rcf for 10 minutes at 4°C. Supernatants were collected and cleared using 0.1 μm pore size centrifugal spin Filters (Millipore Sigma) with centrifugation at ~17,000 rcf for 1 minute at 4°C. From the cleared supernatant, 50 μl were loaded onto glass tubes (SUN-SRi™ Glass Microsampling tubes, ThermoFisher Scientific) for running into a liquid chromatography-mass spectrometer (LC-MS) (Agilent G1956B MSD). Analytes were separated using Synergi 4m, Hydro-RP 80Å, 2.1 x 150mm, 4 mm (Phenomenex), and NAD^+^ was selectively detected using negative polarity with SIM (Selective Ion Monitoring) mode at 662.09 m/z. Here, mobile phases A and B were 4 mM Dibutylammonium acetate pH 5.21 and acetonitrile, respectively. The gradient was starting with 2% of B and changed as follows: 20 min at up to 82%, 1 min at up to 98%, 98% B was held for 3 min and then back to the initial setting within 1 min and re-equilibrated the column for 10 min at 2%. The flow rate maintained for the sample running was 0.2 ml/min. NAD^+^ and BMP were detected at 260 nm and all data were analyzed using Agilent software, ChemStation B.04.03 (Agilent Technologies) (34). The peak height ratio of NAD^+^/BMP was used to calculate NAD^+^ level in each sample.

### Human CD4^+^ T cell isolation.

Primary peripheral blood mononuclear cells (PBMCs) were isolated from human blood by density centrifugation using Lymphocyte Separation Medium (MP Biomedicals). CD4^+^ T cells were negatively selected from PBMCs using the CD4^+^ T Cell Isolation Kit (Miltenyi Biotec) and cultured in RPMI 1640 media supplemented with 25 mM HEPES, 10% heat-inactivated FBS, and Penicillin/Streptomycin. Human CD4^+^ T cells were activated with plate-coated aCD3 (5-10 mg/ml, BioLegend) and aCD28 (10 mg/ml, BioLegend) for 3 days in the presence of 10% autologous serum from the subjects and 5% FBS. Also, CD4^+^ T cells were differentiated into four T cell subtypes by incubation with the specific supplements for Th1, Th2, psoriatic Th17 (30 ng/ml IL-6, 2 ng/ml TGF-b1, 50 ng/ml IL-1b, 20 ng/ml IL-23, 5 mg/ml aIL-4, and 5 mg/ml aIFNg), and Treg, respectively. For Th17 pathogenic specificity, CD4+ T cells were differentiated with the non-pathogenic (NP) differentiation supplements (30 ng/ml recombinant IL-6, 2 ng/ml recombinant TGF-b1, and 5 mg/ml aIFNg antibody) and pathogenic (P) differentiation supplements (30 ng/ml recombinant IL-6, 20 ng/ml recombinant IL-23, and 50 ng/ml recombinant IL-1b). They were differentiated for 3 days on plate-coated TCR (T-cell receptor: aCD3 and aCD28) for Th1, Th2, and Th17 and on aCD3 and aCD46 (10 mg/ml, BioLegend) for Treg in the presence of 10% FBS. All differentiation reagents were purchased from Stemcell Technologies (differentiation supplements of Th1, Th2, and Treg), Peprotech (recombinant proteins), and eBioscience (antibodies).

### Skin biopsy and primary fibroblasts isolation.

Primary fibroblasts are isolated from a skin biopsy by taking a sterile skin sample, typically a 3-mm punch biopsy (Supplemental Figure 7A). Then, human skin biopsies were cut into several pieces, were digested for 30 mins at 37°C in a solution composed of 1 mg/ml Collagenase type II (Invitrogen), 0.25 U/ml Dispase (Invitrogen), and then were incubated for another 30 mins with 7.5 mg/ml DNase (Sigma). After dissociating skin tissues into individual cells in an enzymatic digestion, skin cell samples were filtered using a Cell Strainer of 100 μm (Fisher Scientific) and washed in DMEM. The skin tissue samples were placed under a glass coverslip, cultured with DMEM supplemented with 10% FBS and 100 U/mL Penicillin/Streptomycin (ATCC), and cryopreserved for future experiments. After thawing the fibroblast, the cells were cultures in the Fibroblast growth medium (Sigma) with 2.5% FBS. For investigating the immune response of the fibroblasts to psoriatic Th17 ex vivo components, 5 × 10^6^ cells were incubated with Th17 differentiation supplements (30 ng/ml IL-6, 2 ng/ml TGF-b1, 50 ng/ml IL-1b, 20 ng/ml IL-23, 5 mg/ml aIL-4, and 5 mg/ml aIFNg) for 24 hrs.

### Chemical treatment.

NR (0.5 mM from ChromaDex) and recombinant human SLIT2-N (200 ng/ml from PeproTech and R&D Systems, Endotoxin level: <0.10 EU per 1 μg of the protein) was incubated for 3 days during TCR activation or psoriatic Th17 differentiation. To blocking the immunomodulatory effect of SLIT2, 600 ng/ml of recombinant soluble ROBO1 Fc chimera protein (R&D Systems, Endotoxin level: <0.10 EU per 1 μg of the protein) was treated with vehicle or SLIT2. CDC42 inhibitor ML141, Rac inhibitor Ehop-016, or Rho inhibitor Rhosin was used in cell culture at 20 mM, 10 mM, or 30 mM for 24 hrs, respectively. 10 mM Y27632 (ROCK1/ROCK2 inhibitor) or 10 mM Fasudil (ROCK2 inhibitor) was used for overnight incubation before harvesting the cells. Inhibitors were purchased from Selleck Chemicals and Tocris.

### Cytokine and chemokine assay (ELISA and Multiplex).

Supernatants were collected, centrifuged to remove cells and debris, and stored at −80°C. The levels of cytokines, including IFNg, IL-4, and IL-17 for T cells and IL-6 and TNFa for fibroblasts were measured by ELISA (R&D Systems). Results were normalized to cell number using the CyQuant cell proliferation assay (Invitrogen) or BCA protein assay (Pierce). Also, systemic cytokine assay on the primary fibroblasts inflammation was measured by the human essential immune response panel (13-plex) which is a fluorescence-encoded bead-based multiplex assay (BioLegend). Data were acquired with LSR Fortessa (BD) and post-acquisition analysis was performed using a LEGENDplex data analysis software suite (BioLegend).

### SLIT measurement in the serum.

To collect serum from a red-top tube, draw whole blood into the tube, gently invert it to mix the clot activator, and then let it clot at room temperature for 1 hr. After clotting, serum was collected and stored at −80°C. SLIT1, SLIT2, and SLIT3 in the serum was assayed using the Sandwich ELISA Kits (LSBio).

### RNA-seq library preparation and sequencing.

Total RNA from CD4^+^ T cells at baseline and after four weeks of NR supplementation were extracted with the miRNeasy Micro Kit (Qiagen) and RNA quality and integrity was assessed by Qubit 3.0 fluorometer (Thermo Fisher Scientific) and Agilent Bioanalyzer (Agilent Technologies). Libraries were prepared using TruSeq stranded Total RNA HT kit (Illumina) and sequenced in a HISeq 3000 (Illumina) by the DNA Sequencing and Genomics Core at NHLBI.

### RNA-seq bioinformatics.

RNA sequencing data was processed using RNA-Seek workflow (https://github.com/skchronicles/RNA-seek and https//zenodo.org/records/13312679). This includes extensive preliminary QC: FastQC (v0.11.1) to check read quality, FastQ screen (v0.14.0) and Kraken (v2.0.8-beta) to detect contamination, Cutadapt (v1.18) to remove adapters, low-quality and contaminating reads. Clean reads were mapped to Gencode human genome reference (Release 30 (GRCh38.p12) using STAR (v2.7.6a) aligner in ‘two-pass’ mode. RSEM (v1.3.0) was used to generate gene expression counts. All downstream analyses were performed in R (https://www.R-project.org/). Samples with low gene coverage were removed from RSEM estimated counts matrix using ‘filterByExpr’ function in EdgeR (v4.6.3) (35). The RNA count matrix was prepared using ‘voomWithQualityWeights’ function for differential gene expression analyses in Limma (v3.64.1) (36). The intra-block correlation was applied using ‘duplicateCorrelation’ function in Limma using a vector of patient ID’s as the blocking variable. A linear model was then fit to the data using ‘lmFit’ function in Limma using the same block argument and correlation output from above. Differential gene expression analysis was conducted in Limma by making contrasts (‘makeContrasts’), a linear model was fit to contrasts (‘lmFit’), and statistics were computed using empirical Bayes moderation (‘eBayes’). Benjamini Hochberg (BH) adjustment was applied to differential expression results for multiple hypothesis testing. Genes with nominal *p* value <0.05 were considered differentially expressed (DE) genes. Unfiltered pre-ranked gene list was created using formula: -log(p-value) * sign(logFC), and used as input for Gene Set Enrichment Analysis (GSEA). GSEA was performed using Reactome Pathway Database in the fgsea library (v1.34.2) (37), and using Gene Ontology gene sets with the ‘gseGO’ function in the clusterProfiler library (v4.16.0) (38). BH adjustment was also applied to GSEA results. The pathway enrichment plots, PCA plots and Volcano plots were generated using ggplot2 library in R. The heatmaps were generated using ComplexHeatmap library in R.

### Flow cytometric analyses.

A 16-color antibody panel was designed to enable immunophenotyping of CD4^+^ T cells (Supplemental Table 4, Upper panel). Frozen CD4^+^ T cells were thawed in FACS buffer (PBS with 0.25 mM EDTA and 0.1% BSA) with nuclease (Sigma) and fresh CD4^+^ T cells were isolated from healthy volunteers. The cells were activated with Cell stimulation cocktail plus protein transport inhibitors (eBioscience) and PMA (500 ng/ml, Sigma) for 4 hrs. The antibodies were stained followed by LIVE/DEAD Fixable Yellow stain (Invitrogen). Data were acquired with FACSymphony (BD) and post-acquisition analysis was performed using Flowjo10 (Treestar Inc.). Analysis excluded debris and doublets using light scatter measurements, and dead cells by live/dead stain. The cells were first gated for singlets (FSC-H *ve*rsus FSC-A) and further analyzed for their uptake of the Live/Dead Yellow stain to determine live *versus* dead cells in CD4^+^ T. T cell phenotyping was then determined by double positive cell population within this gated population: Th1 (TBX21^+^IFNg^+^), Th2 (GATA3^+^IL-4^+^), Th17 (RORC^+^IL-17^+^), and Treg (FOXP3^+^CD25^+^). Aditionally, pathogenic T cell phenotyping in live CD4^+^CD161^+^CCR6^+^ gate was determined after differentiation with the non-pathogenic (NP) differentiation supplements and pathogenic (P) differentiation supplements in CD4^+^ T cells (Supplemental Table 4, Lower panel). The gating strategy of flow cytometry was shown in Supplemental Table 5. ROBO1 antibody and isotype mouse IgG was purchased in R&D Systems.

### Gene knockdown experiments.

SMARTpool Accell siRNA was utilized targeting ROBO1, SLIT1, TAGAP, and SRGAP2 (Horizon Discovery). Accell siRNA was transfected with Accell siRNA delivery media according to manufacturer’s instruction (Horizon Discovery). CD4^+^ T cells (4 × 10^6^) with 1.5 μM siRNA were activated with plate-coated TCR for 3 days before assay. Also, primary fibroblasts were transfected with ROBO1 siRNA by Amaxa 4D nucleofector (Lonza) by using P3 primary cell HT nucleofector solution (CA137-AA program). The transfected cells were cultured in Fibroblast growth medium 2 (Sigma) with 2.5% FBS for 48 hrs.

### RNA isolation and quantitative PCR.

Total RNA was extracted using NucleoSpin RNA kit (Macherey-Nagel) and cDNA was synthesized with the SuperScript III First-Strand Synthesis System for RT-PCR (Thermo Fisher Scientific). Quantitative real-time PCR was performed using FastStart Universal SYBR Green Master (Roche) and run on LightCycler 96 Systems (Roche). The primers of canonical transcription factors (TFs) and EF1a of CD4^+^ T cell were made by Integrated DNA Technologies and other targets were measured using validated gene-specific QuantiTech primers (Qiagen - Supplemental Table 6). Relative gene expression was quantified by normalizing cycle threshold values with 18S rRNA and b-Actin using the 2^-ΔΔCt^ cycle threshold method.

### Immunoblot analyses.

Human CD4^+^ T cells were lysed using RIPA buffer supplemented with protease inhibitor cocktail (Roche) and phosphatase inhibitors (Pierce). Lysates were separated by NuPAGE 4%–12% Bis-Tris Protein Gels (Thermo Fisher Scientific) and transferred to nitrocellulose membranes (Bio-Rad Laboratories). Membranes were blocked with Odyssey Blocking Buffer (Li-Cor) and incubated with appropriate antibodies overnight at 4°C. Antibodies were purchased from Cell Signaling Technologies (STAT3, phospho-STAT3 (Tyr705), P70S6K, phospho-P70S6K (Thr389), S6, phospho-S6 (Ser235/236), HA, and Rho-GTPase Antibody Sampler Kit), Proteintech (ROBO1, Myc), Abcam (ROBO1, TAGAP), Sigma (Flag), and Millipore (ACTIN). The secondary antibody conjugated with IRDye 800CW or IRDye 680RD (Li-Cor) were then incubated for 1 hour at room temperature. Immunoblots were scanned using an Odyssey Clx/Dlx imaging system (Li-Cor Biosciences). Protein band intensity was quantified using Image studio software (version 6.1) and normalized to Actin level. Detailed antibody information described in Supplemental Table 7.

### Immunoprecipitation analyses.

293 cells (ATCC) were transfected with Vector (pcDNA3.1), pcDNA-Flag tag-pcDNA-ROBO1 or pReceiver-HA tag-TAGAP (GeneCopoeia) using a Polyjet *in-vitro* DNA transfection reagent (SignaGen Laboratories). The cells were harvested using the lysis buffer (50 mM Tris-HCl, pH 7.4, 1% Triton X-100, 0.5% NP-40, and 0.5 M NaCl) containing protease inhibitor cocktails and phosphatase inhibitors. Protein extract was incubated with anti-Flag M2- or HA-Magnetic beads (Sigma), or HA antibody (Cell Signaling Technologies) and Sera-Mag Speedbeads Protein A/G particles (Sigma) overnight at 4°C. The magnetic beads were washed with lysis buffer and boiled in sample loading buffer. The supernatant was collected for immunoblot using the magnet stand. ROBO constructs were supplied by Dr. Giada Bianchi (Harvard Medical School, Boston, USA) and Dr. Fang-Jen S. Lee (College of Medicine, National Taiwan University, Taipei, Taiwan).

### Rho GTPase activation assay.

Active Rho protein from CD4^+^ T cells was measured by RhoA G-LISA GTPase Activation Assay Kit (Cytoskeleton). The protein extracts were prepared from CD4^+^ T cells (5×10^6^) and quantified using Precision Red advanced protein assay reagent (Cytoskeleton). Same amount of T cell extracts were incubated in the Rho GTP-binding protein linked to the well called G-lisa. Specifically active GTP-bound Rho protein was recognized by an anti-RhoA primary antibody and was measured as a HRP colorimetric readout at OD 490 nm. Constitutively active RhoA protein in the kit was used for positive control. Also active Rho Pull-Down and Detection Kit (Thermo Scientific) was used for monitoring Rho small GTPase activation. Total protein extracts were prepared from CD4^+^ T cells (2×10^7^) and quantified using the BCA protein assay (Pierce). 500 mg protein lysate were incubated with a GST-fusion protein of the Rhotekin-binding domain (RBD) along with glutathione agarose resin. GST-RBD specifically pull down active Rho protein and detected by an anti-Rho antibody for immunoblot. Also two control nucleotides, GTPγS and GDP, were used to generate positive and negative control, respectively.

### Statistics.

Statistical analysis was performed using Prism 7 software (GraphPad) and results are represented as mean ± SEM unless otherwise indicated. Comparisons of 2 groups were calculated using paired or unpaired 2-tailed Student’s *t* tests. Comparisons of more than 2 groups were calculated using 1-way analysis of variance (ANOVA) followed by multiple comparisons test (Sidak or Tukey). Two-way ANOVA was used if there were 2 independent variables. For all tests, *P* < 0.05 was considered significant. For in vivo and ex vitro studies, *n* represents the number of biological replicates per group and is reported in the figure legends.

### Study approval.

The Placebo and NR pilot study was registered in ClinicalTrials.gov with the registration number NCT04271735, approved by NHLBI IRB, and performed at the National Institutes of Health (NIH) Clinical Center. The blood from healthy volunteers for functional study were obtained from participants that consented to enroll on the Disease Discovery Protocol (NCT01143454) and from the NIH Clinical Center blood bank (NCT00001846). To explore the NR effect on primary fibroblasts, skin biopsies from mild to moderate severity psoriasis participants were recruited and consented on (ClinicalTrials.gov registration number NCT01778569) and matched controls from a Disease Discovery Protocol (NCT01143454) respectively.

### Data availability.

All data in the article are included in the Supporting Data Values file, Supplemental Tables, and Supplemental Data. The RNA-seq dataset supplied here in Supplemental Data 1 and 2, will be deposited in the NCBI Gene Expression Omnibus (GEO repository (GSE314390)).

## Author contributions

KH and MNS conceived and designed the project. KH, RJK, TCR, SH, ACR, and PKD performed the experiments. BF and NRR conducted the bioinformatics analysis. RDH recruited the study subjects. KH, RJK, TCR, ACR, RS, AKG, BF, NRR, and PKD provided methodological and analysis support. The manuscript was written by KH and MNS and edited by RJK, TCR, RS, and AKG.

## Conflict of interest

No financial conflict of interests exist. The NR, matching placebo, and the chemical powder were supplied by Chromadex Inc. Los Angeles, California, USA, under a Clinical Cooperative Research and Development Agreement (NIH – CTCR-19-004). No funding for the research was supplied by Chromadex. All of the authors are supported by the Intramural Research Program of the National Institutes of Health (NIH). The contributions of the NIH author(s) were made as part of their official duties as NIH federal employees, are in compliance with agency policy requirements, and are considered Works of the United States Government. However, the findings and conclusions presented in this paper are those of the author(s) and do not necessarily reflect the views of the NIH or the U.S. Department of Health and Human Services.

## Funding support

This work is the result of NIH funding, in whole or in part, and is subject to the NIH Public Access Policy. Through acceptance of this federal funding, the NIH has been given a right to make the work publicly available in PubMed Central.

- The NHLBI Division of Intramural Research (HL005102 – MNS).

## Supplementary Material

Supplemental data

ICMJE disclosure forms

Supplemental data sets 1-4

Unedited blot and gel images

Supporting data values

## Version 1 04/28/2026

In-Press Preview

## Version 2 06/22/2026

Electronic publication
