# Supplementary material for: NAD+ augmentation by nicotinamide riboside engages SLIT2/ROBO1 signaling to attenuate Th17 inflammation in psoriasis
Source: JCI Insight. 2026 Apr 28;11(12):e203826. doi: 10.1172/jci.insight.203826 (PMC13313537; doi:10.1172/jci.insight.203826)
Supplement: Supplemental data [file jciinsight-11-203826-s177.pdf]

SUPPLEMENTAL ONLINE MATERIALS

NAD$^+$ augmentation by nicotinamide riboside engages SLIT2/ROBO1 signaling to attenuate Th17 inflammation in psoriasis

Kim Han,[1] Rachael J. Klein,[1] Thomas C. Recupero,[1] Anna Chiara Russo,[1] Rahul Sharma,[1] Anand K. Gupta,[1] Shahin Hassanzadeh,[1] Rebecca D. Huffstutler,[2] Pradeep K. Dagur,[3] Bryan Fisk,[4] Neelam Redekar,[4] and Michael N. Sack[1,2]

[1]Laboratory of Mitochondrial Biology and Metabolism, NHLBI, NIH, Maryland, USA

[2]Cardiovascular Branch, NHLBI, NIH, Maryland, USA

[3]Flow Cytometry Core Facility, NHLBI, NIH, Maryland, USA

[4]Integrated Data Science Section, NIAID, NIH, Bethesda, MD, USA

Correspondence:

Michael N. Sack, Laboratory of Mitochondrial Biology and Metabolism, NHLBI, NIH,

Bldg. 10-CRC, Room 5-3342, 10 Center Drive, Bethesda, MD 20892, USA

Email: sackm@nih.gov

SUPPLEMENTAL ONLINE MATERIALS

- **Supplemental Figures and Legends (7)**
- **Supplemental Tables (7)**
- **Supplemental Methods**
- **Additional Supplemental Dataset (4) - Uploaded separately as Excel files**

Supplemental Dataset 1: Differential expression genes for comparison of NR supplementation *vs*. Baseline in TCR-activated $CD4^+$ T cells, related to Figure 2

Supplemental Dataset 2: Differential expression genes for comparison of NR supplementation *vs*. Baseline in Naive $CD4^+$ T cells, related to Supplemental Figure 2

Supplemental Dataset 3: GSEA pathway analysis for comparison of NR supplementation *vs*. Baseline in TCR-activated $CD4^+$ T cells, related to Figure 2

Supplemental Dataset 4: GSEA pathway analysis for comparison of NR supplementation *vs*. Baseline in Naive $CD4^+$ T cells, related to Supplemental Figure 2

Supplemental Figures and Legends

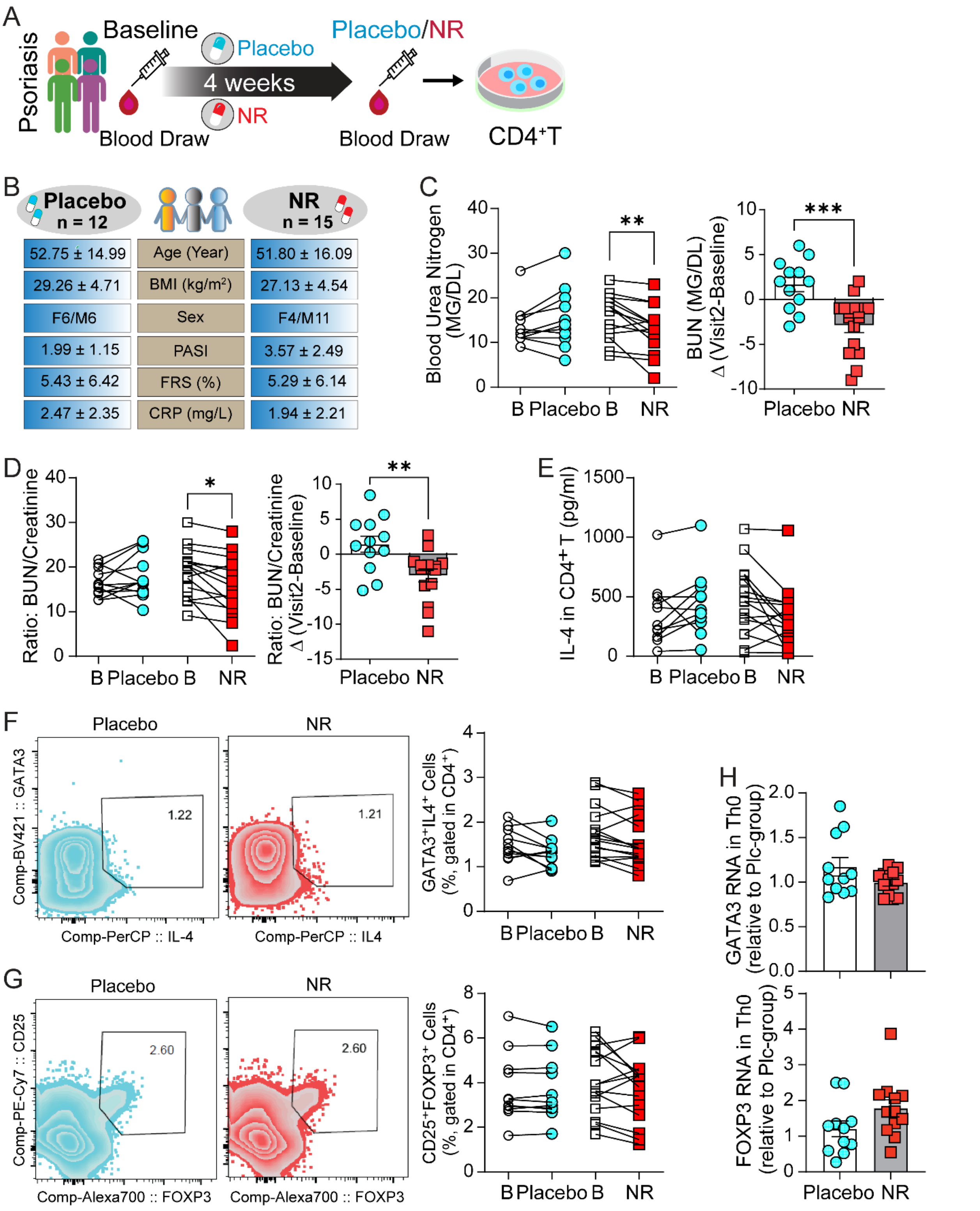

Supplemental Figure 1. Blood biochemistry and CD4⁺ T cell immunophenotyping in NR-supplemented psoriasis participants. (A) Flow diagram of the randomized, placebo-controlled

study in psoriasis participipants reveiving oral NR or placebo for 4 weeks (ClinicalTrials.gov Identifier: NCT04271735). Blood draws at baseline and 4 weeks for $CD4^+$ T cell isolation. (**B**) Participant demographics of placebo and NR groups. Subjects had mild-to-moderate psoriasis (PASI < 12). PASI, psoriasis area and severity index; FSR, fractional synthetic rate; CRP, C-reactive protein. (**C–D**) Delta changes (visit 2 – baseline) in blood urea nitrogen (BUN) and BUN/creatinine ratio in psoriasis subjects (Placebo, n = 12; NR, n = 15). (**E**) IL-4 release in activated $CD4^+$ T cells (Placebo, n = 11; NR, n = 15). (**F–G**) Flow cytometry plots showing Th2 ($IL\text{-}4^+GATA3^+$) and Treg ($CD25^+FOXP3^+$) subsets. (**H**) *GATA3* and *FOXP3* mRNA expression normalized to *EF1α*. Data are mean ± SEM. *P* values determined by paired or unpaired two-tailed Student's *t*-test. *$p<0.05$, **$p<0.01$, ***$p<0.001$.

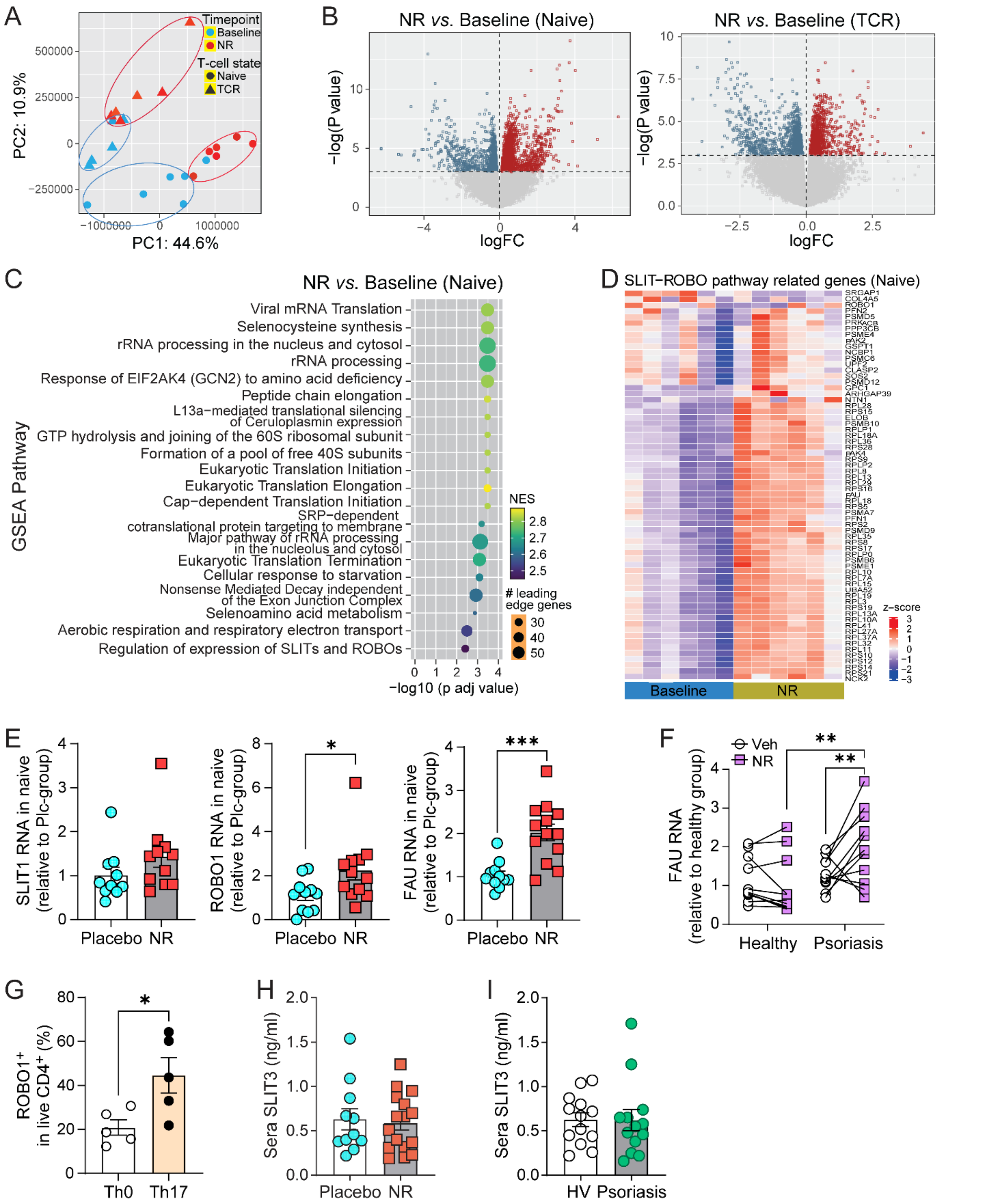

Supplemental Figure 2. RNA-seq analysis of naïve psoriatic CD4^{+} T cells following *in vivo* NR supplementation. (A) Principle component analysis (PCA) generated using all genes detected in NR and baseline under naïve or activated CD4^{+} T cell state. The x and y axis represents PC1 (44.6% explained variance) and PC2 (10.9% explained variance), respectively - suggesting,

primary and secondary sources of variation in gene expression data is contributed by timepoint and T-cell states respectively. **(B)** Volcano plots showing all the genes from NR *vs.* baseline comparisons under naïve or activated $CD4^+$ T cell state of psoriatic subjects (n=5-6). Differentially expressed genes significant at *p* value<0.05 that showed increased expression in NR were highlighted in red (logFC>1) while those that showed decreased expression in NR were highlighted in blue (logFC<-1). **(C)** Top 20 C2 reactome pathway enriched in NR *vs.* baseline comparison in naive psoriatic $CD4^+$ T cells. The x axis represents negative log10 transformed adjusted *p* values from GSEA analysis. (**D**) Heatmap of SLIT–ROBO pathway genes in naïve $CD4^+$ T cells. (**E**) qPCR of *SLIT1*, *ROBO1*, and *FAU* in psoriatic $CD4^+$ T cells from placebo or NR groups (n = 10–12/group). (**F**) *FAU* mRNA expression in healthy vs. psoriatic $CD4^+$ T cells (n = 11/group, ClinicalTrials.gov Identifiers: NCT01778569 and NCT01143454). (**G**) Surface ROBO1 expression in Th0 and Th17. (**H–I**) Serum SLIT3 levels in psoriatic subjects (Placebo, n = 11; NR, n = 15) and healthy volunteers (HV, n = 11). Data are mean ± SEM. *P* values determined by paired or unpaired two-tailed Student's *t*-test. *$p<0.05$, **$p<0.01$, ***$p<0.001$.

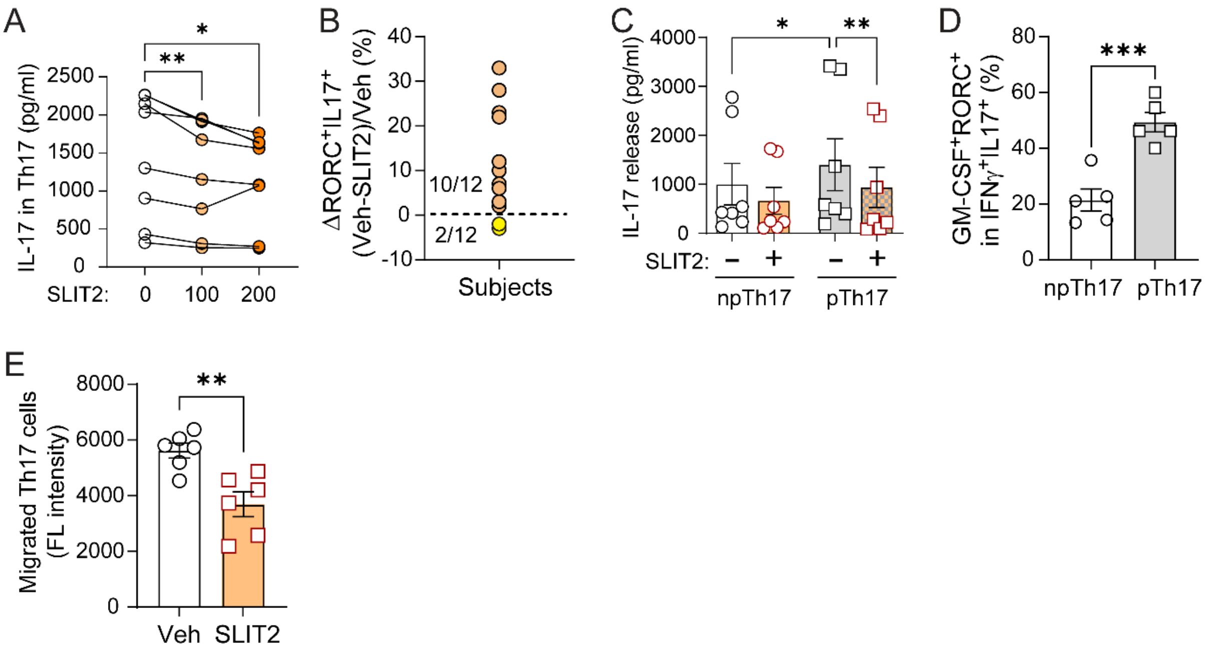

Supplemental Figure 3. SLIT2 decreases IL-17 release and migration of pathogenic Th17 cells. (**A**) IL-17 secretion measured by ELISA in psoriatic CD4^{+} T cells treated with increasing concentrations of SLIT2 (0–200 ng/ml, 72 hrs; n = 8). (**B**) Data are presented as the change (Δ (%) = (Veh−SLIT2)/Veh) from the flow cytometry of Th17 (RORC^{+}IL-17^{+}) population of pathogenic Th17 cells treated with SLIT2 treatment (related to Figure 3D, n = 12). (**C**) IL-17 release in nonpathogenic (np) and pathogenic (p) Th17 cells treated with vehicle or SLIT2 (200 ng/ml; n = 7). npTh17 differentiation: IL-6, TGF-β1, and αIFNγ; pTh17 differentiation: IL-6, IL-23, and IL-1β. (**D**) Flow cytometric analysis of pathogenic Th17 cells, defined as IL-17A^{+}GM-CSF^{+} RORC^{+} cells within the CD4^{+}CD161^{+}CCR6^{+} population, enriched for CD26^{hi} and CCR4^{lo}/TBX21^{+} phenotypes, compared with non-pathogenic Th17 cells (n = 5). (**E**) Migration of psoriatic Th17 cells assessed using a Boyden chamber assay quantified by Hoechst fluorescence (n = 6). Data are mean ± SEM. *P* values determined by paired two-tailed Student's *t*-test or one-way ANOVA for multiple groups. *$p<0.05$, **$p<0.01$.

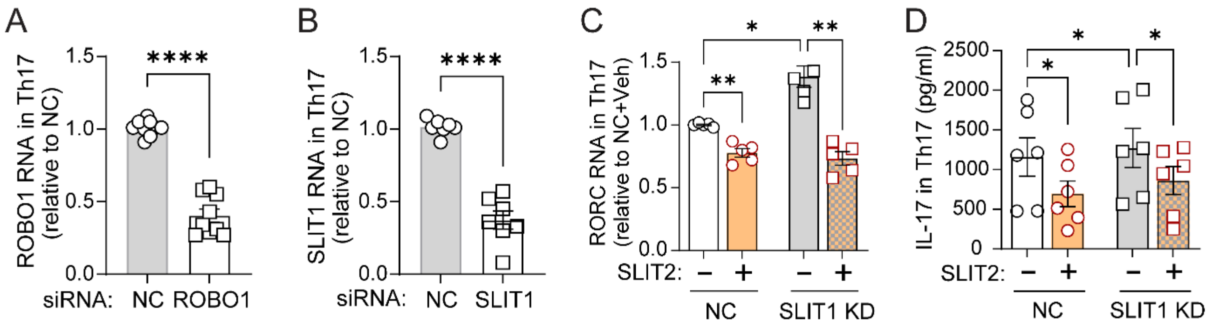

Supplemental Figure 4. SLIT1 knockdown does not affect SLIT2-mediated inhibition of IL-17 release in psoriatic Th17 cells. (**A**) ROBO1 knockdown efficiency in psoriatic Th17 cells transfected with ROBO1 siRNA. (**B**) SLIT1 knockdown efficiency in psoriatic Th17 cells transfected with control or SLIT1 siRNA (n = 7). (**C**) *RORC* mRNA expression in control or SLIT1 siRNA–transfected Th17 cells (n = 5). (**D**) IL-17 secretion measured by ELISA in SLIT1-depleted Th17 cells treated with vehicle or SLIT2 (n = 6). Data are mean ± SEM. *P* values were determined by paired two-tailed Student's *t*-test or one-way ANOVA with multiple comparisons. *p<0.05, **p<0.01, ***p<0.001, ****p<0.0001.

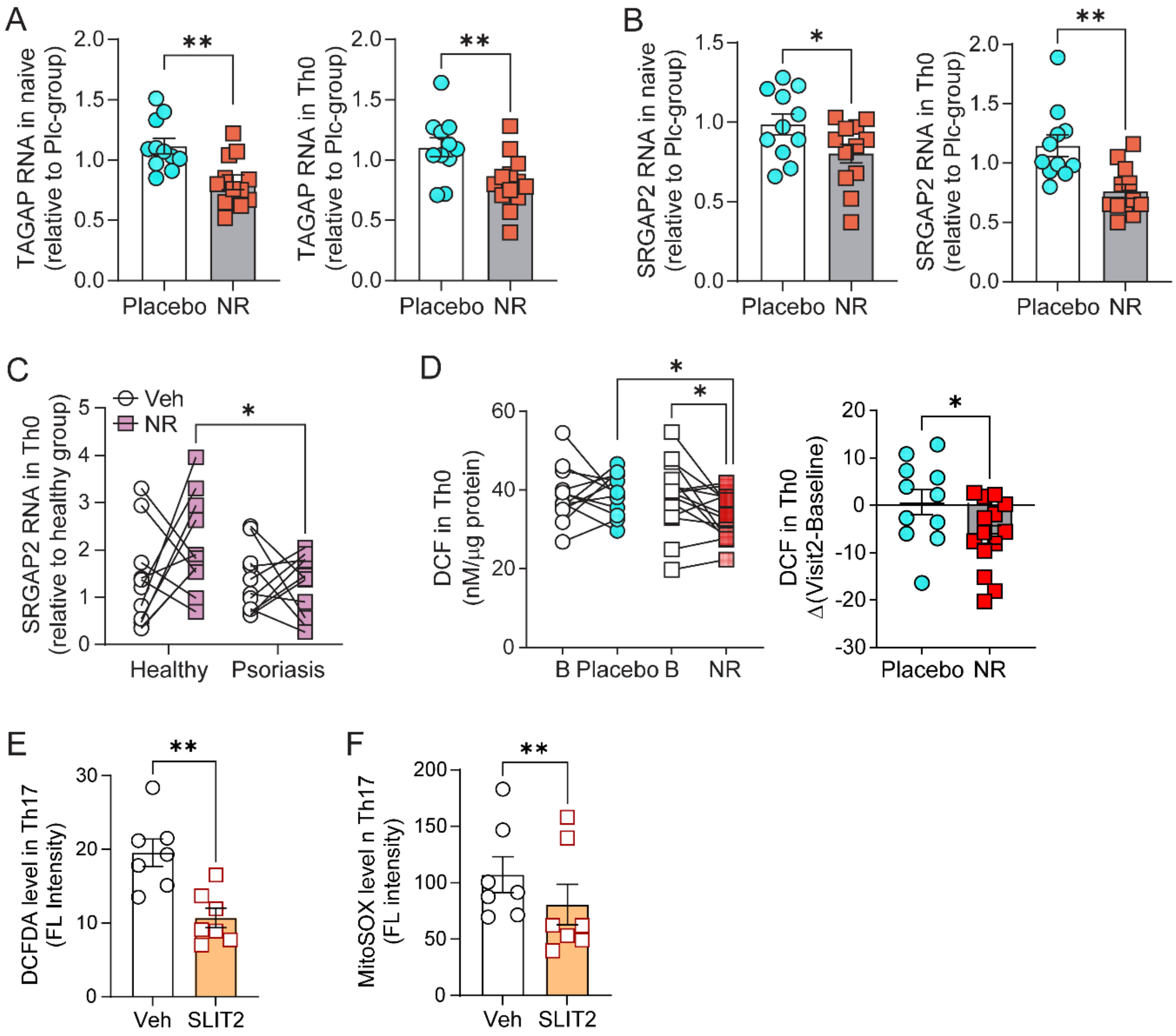

Supplemental Figure 5. SLIT2 attenuates reactive oxygen species (ROS) synthesis in psoriatic CD4^{+} T cells. (**A–B**) Relative mRNA expression of *TAGAP* and *SRGAP2* in placebo and NR groups at visit 2 (Placebo, n = 11; NR, n = 12). Data were normalized to *18S* rRNA or *β-Actin*. (**C**) *SRGAP2* expression in naïve and activated CD4^{+} T cells from psoriatic individuals receiving placebo or NR (500 mg daily for 4 weeks; n = 10–12/group). (**D**) ROS/RNS (reactive nitrogen species) activity in whole blood from psoriatic subjects after placebo or NR supplementation (Placebo, n = 11; NR, n = 12). (**E–F**) Cellular ROS (DCFDA staining) and mitochondrial ROS (MitoSOX staining) levels measured by flow cytometry in psoriatic Th17 cells treated with vehicle or SLIT2 (n = 7). Data point of each subject were shown as dot and all data were represented as mean ± SEM. The Statistical analyses of experiments were performed for comparisons of two groups using unpaired or paired two-tailed Student's *t*-test. One-way ANOVA analysis was followed by multiple comparisons test. *p<0.05, **p<0.01, ***p<0.001, ****p<0.0001.

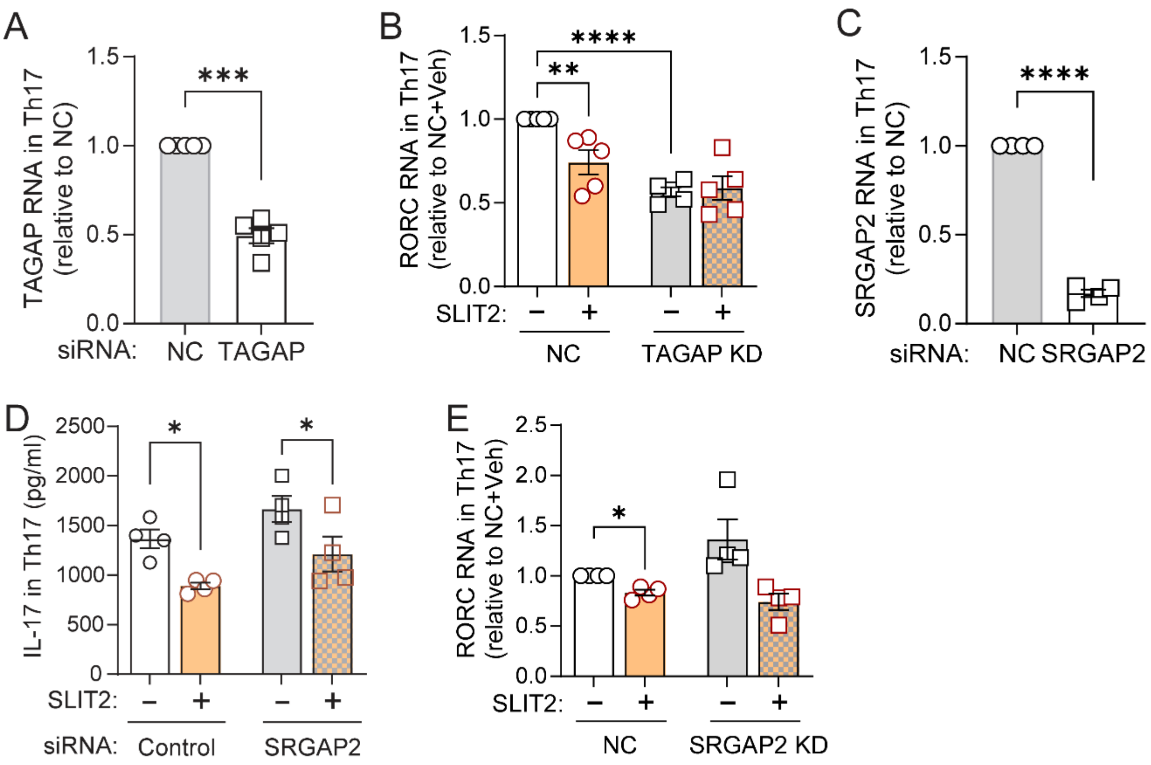

Supplemental Figure 6. SRGAP2 does not modulate SLIT2 effects in psoriatic Th17 cells. (**A**) Knockdown efficiency of *TAGAP* in psoriatic Th17 cells transfected with TAGAP siRNA. (**B**) Relative mRNA expression of *RORC* in Th17 cells transfected with control or TAGAP siRNA (n = 5). (**C**) Knockdown efficiency of *SRGAP2* in psoriatic Th17 cells transfected with control or SRGAP2 siRNA. (**D**) IL-17 production measured by ELISA in SRGAP2-depleted Th17 cells treated with vehicle or SLIT2 (n = 6). (**E**) Relative mRNA expression of *RORC* in Th17 cells transfected with control or SRGAP2 siRNA (n = 4). Data are mean ± SEM. The *p* values for comparisons of two groups were calculated using paired two-tailed Student's *t*-test (Veh *vs*. SLIT2 or NC *vs*. KD). One-way ANOVA analysis was followed by multiple comparisons test. **p*<0.05, ***p*<0.01, ****p*<0.001, *****p*<0.0001.

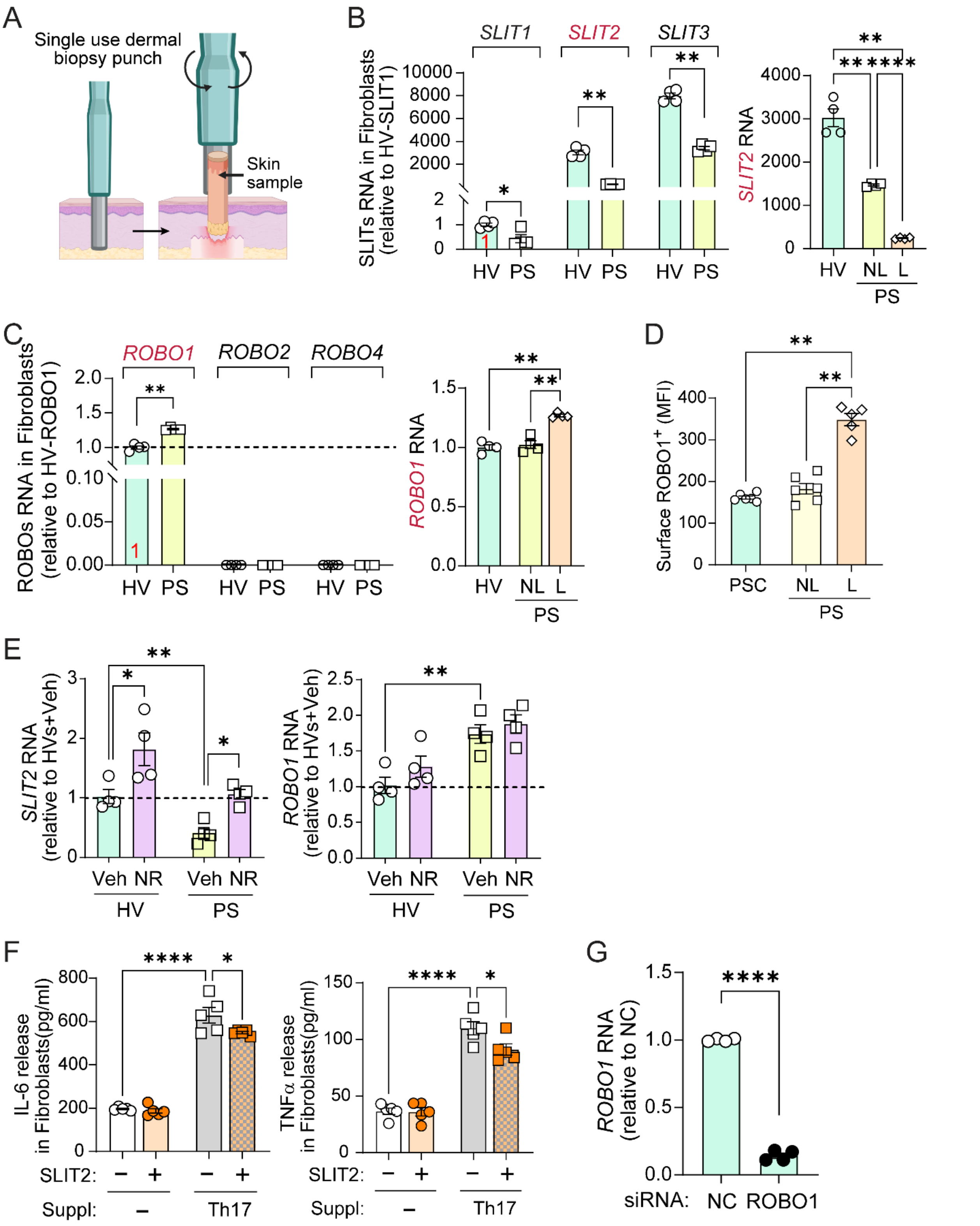

Supplemental Figure 7. SLIT2 reduces inflammatory responses in fibroblasts from psoriasis subjects. (**A**) Skin punch biopsies were collected using a round cutting device; psoriasis biopsies were obtained from lesions. (**B-C**) Relative mRNA expression of *SLIT* and *ROBO* genes in fibroblasts from healthy volunteers (HV) and from non-lesion (NL) and lesion (L) skin of psoriasis

subjects (PS) (n = 4 per group). (**D**) Surface expression of ROBO1 in fibroblasts from healthy volunteers and psoriasis subjects (n = 5 per group). (**E**) Relative mRNA expression of *SLIT2* and *ROBO1* in HV and PS fibroblasts treated with NR (n = 5). (**F**) IL-6 and TNFα secretion from HV fibroblasts incubated with Th17 differentiation conditioned media (n = 5). (**G**) Knockdown efficiency of *ROBO1* in fibroblasts transfected with ROBO1 siRNA. All data were represented as mean ± SEM. The *p* values for comparisons of two groups were calculated using unpaired two-tailed Student's *t*-test (HV *vs*. PS) or paired two-tailed Student's *t*-test (Veh *vs*. NR, Veh *vs*. SLIT2, or NC *vs*. KD). One-way ANOVA analysis was calculated for the multiple groups. *$p<0.05$, **$p<0.01$, ***$p<0.001$, ****$p<0.0001$.

Supplemental Tables

Supplemental Table 1. Complete blood count (CBC) with differential in psoriatic subjects following 4-week *in vivo* placebo or NR supplementation (Clinical Trian No.: NCT04271735).

**Blood Cell Type**	**Visit**	**Placebo Group**	**NR Group**	***P v*alue** (Placebo *vs*. NR)
Subjects number		12	14	n/a
**Absolute count (K/MCL)**				
WBC	Baseline	6.25 ± 1.97	5.34 ± 1.03	0.15
	Visit 2	5.95 ± 1.53	5.52 ± 1.10	0.40
Lymphocytes	Baseline	1.65 ± 0.56	1.60 ± 0.29	0.86
	Visit 2	1.66 ± 0.52	1.60 ± 0.34	0.77
Monocytes	Baseline	0.48 ± 0.18	0.47 ± 0.11	0.90
	Visit 2	0.48 ± 0.15	0.50 ± 0.10	0.74
Neutrophils	Baseline	3.93 ± 1.51	3.09 ± 0.99	0.10
	Visit 2	3.60 ± 1.03	2.87 ± 0.64	0.04*
Eosinophils	Baseline	0.14 ± 0.09	0.14 ± 0.07	0.82
	Visit 2	0.15 ± 0.10	0.17 ± 0.10§	0.52
Basophils	Baseline	0.036 ± 0.02	0.036 ± 0.02	0.82
	Visit 2	0.042 ± 0.02§	0.038 ± 0.02	0.61
**Relative Count (%)**				
Lymphocytes	Baseline	27.07 ± 7.85	30.89 ± 8.16	0.24
	Visit 2	28.10 ± 6.24	30.49 ± 7.07	0.38
Monocytes	Baseline	7.53 ± 1.49	8.73 ± 1.53	0.06
	Visit 2	8.15 ± 2.12	9.32 ± 1.29§	0.10
Neutrophils	Baseline	62.10 ± 8.12	56.69 ± 8.42	0.11
	Visit 2	60.28 ± 6.30	54.63 ± 6.22	0.04*
Eosinophils	Baseline	0.14 ± 0.09	0.14 ± 0.07	0.82
	Visit 2	0.15 ± 0.10	0.17 ± 0.10§	0.52
Basophils	Baseline	0.58 ± 0.23	0.68 ± 0.29	0.33
	Visit 2	0.68 ± 0.26§	0.71 ± 0.36	0.81

Data are represented as mean ± standard deviation (SD). Comparisons between two groups were calculated using an unpaired two-tailed Student's t-test (Placebo *vs*. NR; *$p<0.05$) or paired two-tailed Student's *t*-test (Baseline *vs*. Visit 2; §$p<0.05$). CBC, complete blood count; WBC, white blood cell count; K, $\times 10^3$.

Supplemental Table 2. CBC with differential in healthy subjects following 7-day *in vivo* NR (500 mg bid) or placebo supplementation (Clinical Trian No.: NCT02812238) (13).

**Blood Cell Type**	**Visit**	**Placebo Group**	**NR Group**	***P value*** (Placebo *vs.* NR)
Subject No.		12	13	
**Absolute count (K/MCL)**				
WBC	Baseline	5.97 ± 1.41	5.67 ± 1.49	0.61
	Visit 2	6.50 ± 2.75	4.59 ± 1.26§	0.03*
Lymphocytes	Baseline	1.96 ± 0.38	1.59 ± 0.30	0.01
	Visit 2	2.00 ± 0.45	2.20 ± 0.45	0.34
Monocytes	Baseline	0.49 ± 0.16	0.46 ± 0.13	0.71
	Visit 2	0.47 ± 0.19	0.40 ± 0.14	0.31
Neutrophils	Baseline	3.31 ± 1.25	3.45 ± 1.27	0.79
	Visit 2	3.83 ± 2.40	2.20 ± 1.04§§§	0.03*
Eosinophils	Baseline	0.16 ± 0.11	0.10 ± 0.08	0.14
	Visit 2	0.13 ± 0.10	0.11 ± 0.08	0.55
Basophils	Baseline	0.03 ± 0.02	0.04 ± 0.01	0.49
	Visit 2	0.04 ± 0.02	0.04 ± 0.02	0.84
**Relative Count (%)**				
Lymphocytes	Baseline	34.00 ± 7.98	29.75 ± 8.81	0.22
	Visit 2	33.84 ± 10.37	41.68 ± 10.89§§§	0.08
Monocytes	Baseline	8.32 ± 2.17	8.32 ± 1.67	1.00
	Visit 2	7.49 ± 1.89	8.82 ± 2.13	0.11
Neutrophils	Baseline	54.21 ± 8.81	59.03 ± 9.65	0.21
	Visit 2	55.55 ± 11.14	45.63 ± 11.83§§§	0.04*
Eosinophils	Baseline	2.58 ± 1.64	1.73 ± 1.07	0.14
	Visit 2	2.20 ± 1.71	2.38 ± 1.46§	0.78
Basophils	Baseline	0.58 ± 0.27	0.72 ± 0.28	0.24
	Visit 2	0.63 ± 0.31	0.82 ± 0.40	0.20

Data are represented as mean ± standard deviation (SD). Blood from visit2 was collected under 24-hr fasting state. Comparisons between two groups were calculated using an unpaired two-tailed Student's t-test (Placebo *vs*. NR; *p<0.05) or paired two-tailed Student's t-test (Baseline *vs*. Visit 2; §p<0.05, §§§p<0.001). CBC, complete blood count; BMI, body mass index; WBC, white blood cell count.

Supplemental Table 3. Serum biochemistry in psoriatic subjects following 4-week *in vivo* placebo or NR supplementation (Clinical Trian No.: NCT04271735).

**Serum biochemistry**	**Visit**	**Placebo**	**NR**	***P v*alue** (Placebo *vs*. NR)
Subjects number		9	9	n/a
NAD (AU)	Baseline	0.51 ± 0.11	0.55 ± 0.19	0.54
	Visit 2	0.49 ± 0.14	1.36 ± 0.72§§	0.003**
Subjects number		12	15	n/a
Glucose (MG/DL)	Baseline	107.58 ± 28.38	98.80 ± 9.24	0.27
	Visit 2	103.42 ± 13.67	99.60 ± 7.55	0.37
Albumin (G/DL)	Baseline	4.47 ± 0.32	4.39 ± 0.24	0.50
	Visit 2	4.28 ± 0.25	4.37 ± 0.18	0.28
C-reactive protein (MG/L)	Baseline	2.47 ± 2.45	1.80 ± 2.41	0.50
	Visit 2	2.83 ± 2.17	0.92 ± 0.63	0.008**
Alanine Aminotransferase (U/L)	Baseline	25.83 ± 11.31	23.73 ± 10.69	0.63
	Visit 2	24.83 ± 10.31	23.73 ± 11.77	0.80
Alkaline Phosphatase (U/L)	Baseline	73.58 ± 22.77	59.33 ± 15.55	0.07
	Visit 2	73.17 ± 23.15	60.53 ± 14.51	0.10
Aspartate Aminotransferase (U/L)	Baseline	25.17 ± 8.14	23.40 ± 4.34	0.48
	Visit 2	23.40 ± 4.34	24.27 ± 5.84	0.91
Direct Bilirubin (MG/DL)	Baseline	0.23 ± 0.08	0.28 ± 0.09	0.16
	Visit 2	0.22 ± 0.06	0.28 ± 0.08	0.03*
Urea nitrogen (MG/DL)	Baseline	14.00 ± 4.45	16.13 ± 5.03	0.26
	Visit 2	15.67 ± 6.46	13.27 ± 5.55§§	0.31
BUN/Creatinine Ratio	Baseline	16.53 ± 3.04	18.72 ± 5.56	0.23
	Visit 2	17.97 ± 5.00	15.70 ± 6.77§§	0.34
Cholesterol (MD/DL)	Baseline	200.33 ± 51.67	186.33 ± 35.21	0.41
	Visit 2	205.50 ± 50.24	179.93 ± 32.73§§	0.12
HDL Cholesterol (MD/DL)	Baseline	57.00 ± 20.56	53.13 ± 14.29	0.57
	Visit 2	55.50 ± 18.39	51.93 ± 12.53	0.56
Triglycerides (MG/DL)	Baseline	125.83 ± 68.55	103.53 ± 52.03	0.35
	Visit 2	118.27 ± 39.42	101.47 ± 39.78	0.30

Data are represented as mean ± standard deviation (SD). *P* values were calculated using an unpaired two-tailed Student's *t*-test for comparisons between Placebo and NR groups (**p*<0.05; ***p*<0.01) and a paired two-tailed Student's *t*-test for comparisons of Baseline *vs*. Visit 2 within the same group (§§*p*<0.01). Abbreviations: BMI, body mass index; PASI, psoriasis area and severity index; FRS, Framingham risk score; CRP, C-reactive protein; BUN, blood urea nitrogen; n/a, not applicable.

Supplementary Table 4. Human $CD4^{+}$ T cell phenotype antibody panel (17-color, upper) and pathogenic Th17 subset panel (16-color, lower) used for flow cytometry.

Laser Line	Target	Fluorescent dye	Clone	Vendor	Catalog#
355 nm (UV)	TNF-α	BUV395	Mab11	BD	563996
	CD4	BUV496	SK3	BD	612936
	HLA-DR	BUV661	G46-6	BD	612980
	CD8	BUV737	SK1	BD	612755
	CD45	BUV805	HI30	BD	612892
407 nm (Violet)	GATA3	BV421	L50-823	BD	563349
	Live/Dead	BV585		Invitrogen	L34959
	CD38	BV650	HIT2	BD	740574
	CD3	BV711	UCHT1	BD	563725
	IL-2	BV786	MQ1-17H12	BioLegend	500348
488 nm (Blue)	RORC	BB515 Alexa488	Q21-559	BD	563621
	IL-4	Percpcy5.5	8D4-8	BD	561234
562nm (Yellow Green)	TBX21	PE	O4-46	BD	561268
	CD25	PECy7	M-A251	BD	557741
640nm (Red)	IL-17A	APC (Alexa647)	SCPL1362	BD	560437
	FOXP3	APC700	PCH101	eBioscience	56-4776-41
	IFN-γ	APCcy7	4S.B3	BioLegend	502530
**Replaced the antibody for Pathogenic Phenotyping**					
407 nm	GM-CSF	BV421	BVD2-21C11	BD	562930
	CD26	BV650	YU66	BD	744451
	CD161	BV786	HP-3G10	BD	748281
488 nm	CCR4	Percpcy5.5	1G1	BD	560726
562 nm	CCR6	PECy7	11AG	BD	560620
640 nm	No antibody	APC700			

Supplementary Table 5. Flow cytometry gating strategy.

**Human CD4⁺ T Cell Phenotyping**	
**Cell Population**	**Flow Cytometry Gating**
Cells/Singlets/Live CD45+	Live/Dead Yellow-CD45+
CD3+	CD3+CD8-
CD4+	CD3+CD8-CD4+
Th1 cells	CD4+TBX21+IFNγ+
Th2 cells	CD4+GATA3+IL4+
Th17 cells	CD4+RORC+IL17+
Treg cells	CD4+FOXP3+
CD4+: Cytokines	CD4+IFNγ+<br>CD4+IL4+<br>CD4+IL17A+<br>CD4+IL2+<br>CD4+TNFα+
CD4+: Activated	CD4+CD38+
	CD4+HLADR+
	CD4+CD38+/HLADR+
**Human Th17 Pathogenic Phenotyping**	
**Cell Population**	**Flow Cytometry Gating**
Cells/Singlets/Live CD45+	Live/Dead Yellow-CD45+
CD3+	CD3+CD8-
CD4+	CD3+CD8-CD4+
Th17 lineage	CD4+CD161+CCR6+
Pathogenic Th17	CD4+CD161+CCR6+CD26hi
Polarized Th17	CD4+CD161+CCR6+CD26hi CC4loTBX21+
Q1: IFNγ+	IFNγ+
Q2: IFNγ+ IL17+ (Functional Th17)	IFNγ+IL17+ (Th17.1)
GM-CSF+	IFNγ+IL17+GM-CSF+
GM-CSF+RORC+	IFNγ+IL17+GM-CSF+RORC+
Q3: IL17+	IL17+
Q4: IFNγ-IL17-	IFNγ-IL17-

Supplemental Table 6. Custom-designed and pre-made primer sets used for quantitative real-time RT-PCR.

Oligonucleotides	SOURCE	IDENTIFIER
TBX21<br>Fwd: 5' CGTGACTGCCTACCAGAAT 3'<br>Rev: 5' ATCTCCCCCAAGGAATTGAC 3'	This manuscript	N/A
RORC<br>Fwd: 5' GCATGTCCCGAGATGCTGTC 3'<br>Rev: 5' CTGGGAGCCCCAAGGTGTAG 3'	This manuscript	N/A
GATA3<br>Fwd: 5' GAACCGGCCCCTCATTAAG 3'<br>Rev: 5' ATTTTTCGGTTTCTGGTCTGGAT 3'	This manuscript	N/A
FOXP3<br>Fwd: 5' GTGGCATTCAAGGAGTACCTC 3'<br>Rev: 5' TGATGGCCTTCGATTCTGGATT 3'	This manuscript	N/A
EF1a<br>Fwd: 5' GTTGATATGGTTCCTGGCAAGC 3'<br>Rev: 5' GCCAGCTCCAGCAGCCTTC 3'	This manuscript	N/A
SLIT1	Qiagen	QT00071113
SLIT2	Qiagen	QT00007784
SLIT3	Qiagen	QT00018795
SLIT4	Qiagen	QT02564065
ROBO1	Qiagen	QT01668982
ROBO2	Qiagen	QT01007664
ROBO3	Qiagen	QT00055951
ROBO4	Qiagen	QT00237741
SRGAP1	Qiagen	QT00075068
SRGAP2	Qiagen	QT00084637
SRGAP3	Qiagen	QT00077868
TAGAP	Qiagen	QT00091238
TAGAP va.1	Qiagen	QT01029770
RRN18S	Qiagen	QT00199367
β-ACTIN	Qiagen	QT00095431

Supplemental Table 7. Details of the antibodies used for this study.

**Antibody**	**WB Dilution**	**Catalog number**	**RRID**	**Vendor**
STAT3	1:1000	12640	AB_2629499	Cell Signaling
Phospho-STAT3 (Tyr705)	1:1000	9145	AB_2491009	Cell Signaling
P70S6K	1:1000			Cell Signaling
Phospho-P70S6K (Thr389)	1:1000	9205	AB_330944	Cell Signaling
S6	1:1000	2217	AB_331355	Cell Signaling
Phospho-S6 (Ser235/236)	1:1000	4858	AB_916156	Cell Signaling
HA	1:1000	3724	AB_1549585	Cell Signaling
Rho-GTPase Antibody Sampler Kit	1:1000	9968	AB_10693922	Cell Signaling
Myc	1:1000	16286-1-AP	AB_11182162	Proteintech
ROBO1	1:1000	20219-1-AP	AB_10694675	Proteintech
ROBO1	1:1000	ab7279	AB_449561	Abcam
TAGAP	1:1000	ab187664	AB_2922758	Abcam
FLAG	1:1000	F3165	AB_259529	Sigma
ACTIN	1:10000	MAB1501	AB_2223041	Millipore
IRDye800CW Goat anti-rabbit IgG	1:10000	926-32211	AB_621843	Li-Cor Bioscience
IRDye680RD Donkey anti-mouse IgG	1:10000	926-68072	AB_10954628	Li-Cor Bioscience
IRDye680RD Goat anti-rabbit IgG	1:10000	926-68071	AB_10956166	Li-Cor Bioscience

Supplemental Methods

Transwell migration assay.

Migration of psoriatic $CD4^+$ T cells was assessed using 4 μm pore Transwell inserts (Costar). $CD4^+$ T cells (5×10^6) were polarized to Th17 cells for 3 days in psoriatic Th17 differentiation media, then seeded in the upper chamber containing 1% FBS media. The lower chamber contained 400 μg/ml recombinant SLIT2 and 2× soluble TCR (Stemcell Technologies). After 24 hours, migrated cells in the lower chamber were collected, stained with 5 μM Hoechst 33342, and quantified by fluorescence using a microplate reader (Ex. 360 nm/Em. 460 nm).

Intracellular and mitochondrial ROS measurement.

The total free radical presence of $CD4^+$ T cells (25 μg) was measured using OxiSelect *in vitro* ROS (reactive oxygen species)/RNS (reactive nitrogen species) assay kit (Cell Biolabs). The assay employs a proprietary quenched fluorogenic probe, dichlorodihydrofluorescin DiOxyQ (DCFH-DiOxyQ), which is oxidized by ROS/RNS probe that is based on similar chemistry to the popular DCFDA. ROS and RNS species can react with prepared DCFH probe, which is rapidly oxidized to the highly fluorescent DCF. Samples were measured fluorometrically against a DCF standard by microplate reader (Ex. 480 nm/Em. 530 nm). Results were normalized to protein concentration using the BCA protein assay (Pierce).

ROS generation in the form of superoxide was measured also by a microplate reader-based assay using DCFDA (2',7'-Dichlorofluorescin Diacetate, Sigma) for intracellular ROS and MitoSOX (mitochondrial superoxide indicator, Invitrogen) for mitochondrial ROS. $CD4^+$ T cells (1×10^6 per well) were incubated with 10 μM DCFDA or 10 μM MitoSOX in HBSS medium for 30 minutes at 37°C. Cells were subsequently washed with HBSS, and the fluorescence intensities of each subset/cell were measured by flow cytometry (BD LSR Fortessa).
