# Supplementary material for: NAD+ augmentation by nicotinamide riboside engages SLIT2/ROBO1 signaling to attenuate Th17 inflammation in psoriasis
Source: JCI Insight. 2026 Apr 28;11(12):e203826. doi: 10.1172/jci.insight.203826 (PMC13313537; doi:10.1172/jci.insight.203826)

Full unedited blots for Figure 3E

✂- - - - blot precut prior to antibody incubation to follow multiple antibodies per blot

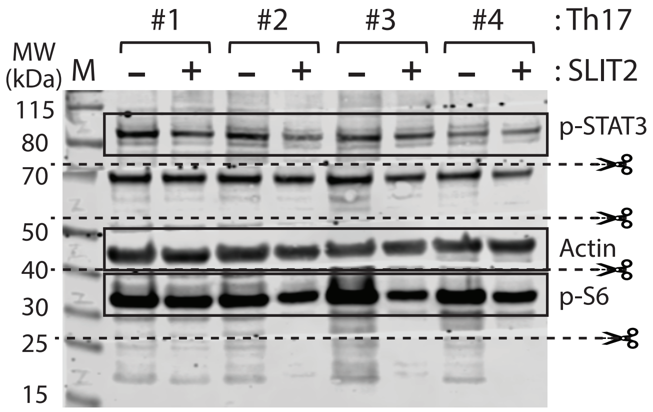

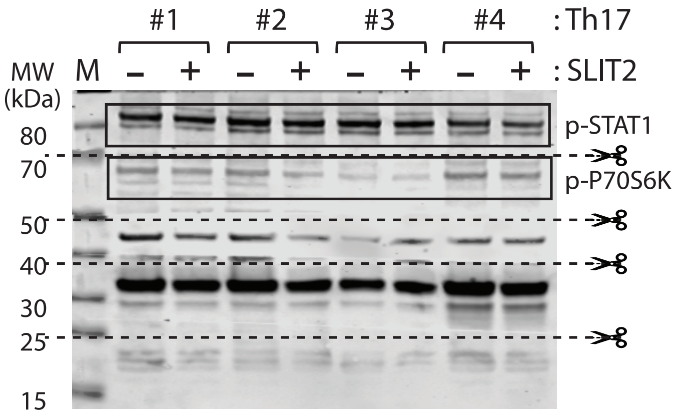

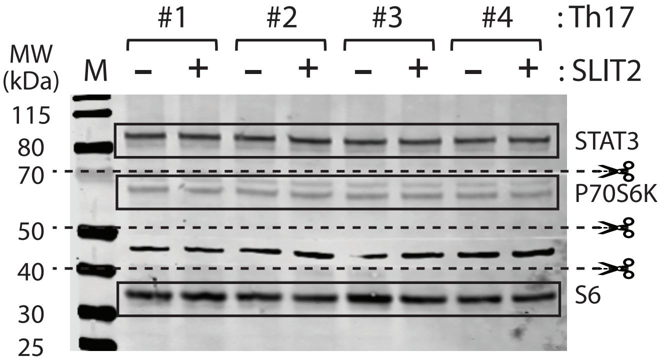

Full unedited blots for Figure 4F

✂- - - - blot precut prior to antibody incubation to follow multiple antibodies per blot

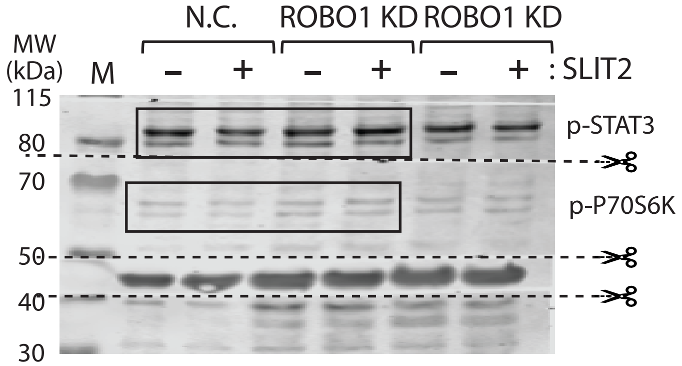

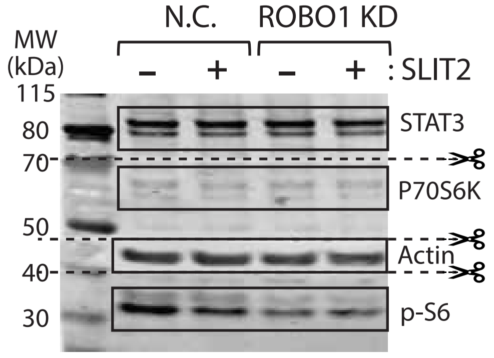

Full unedited blots for Figure 5

Figure 5D

✂- - - - blot precut prior to antibody incubation to follow multiple antibodies per blot

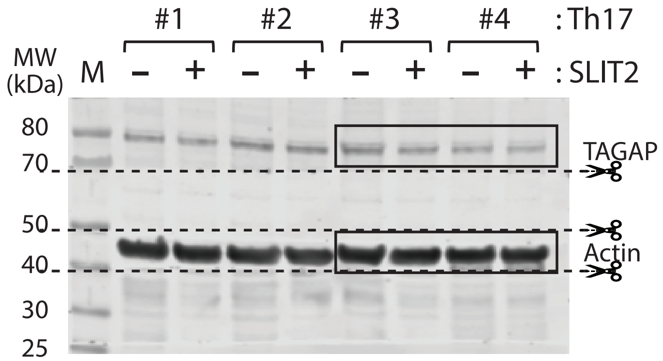

Figure 5H

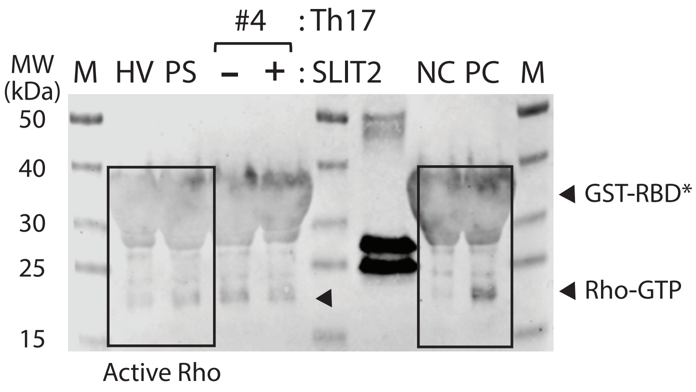

Figure 5I

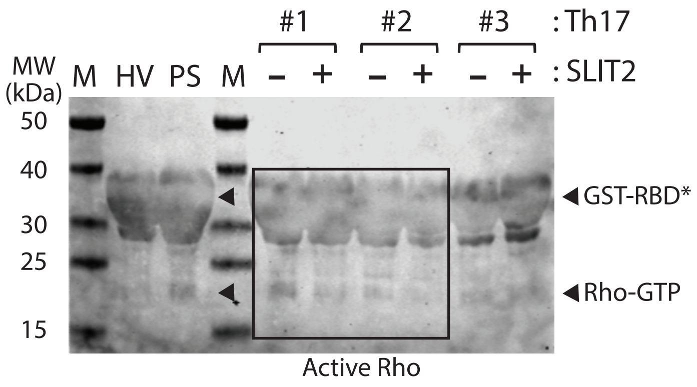

Full unedited blots for Figure 6

- - - - blot precut prior to antibody incubation to follow multiple antibodies per blot

Figure 6E

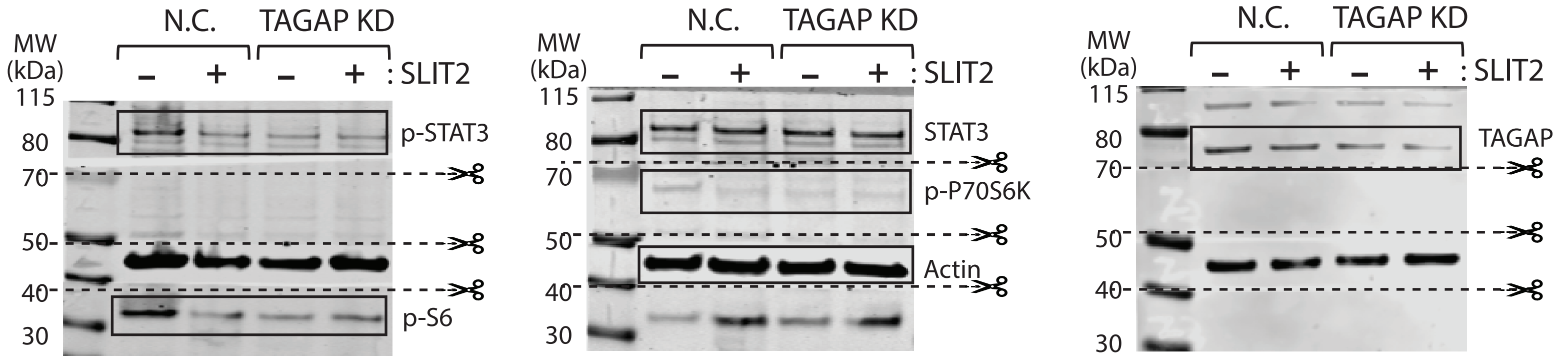

Figure 6G

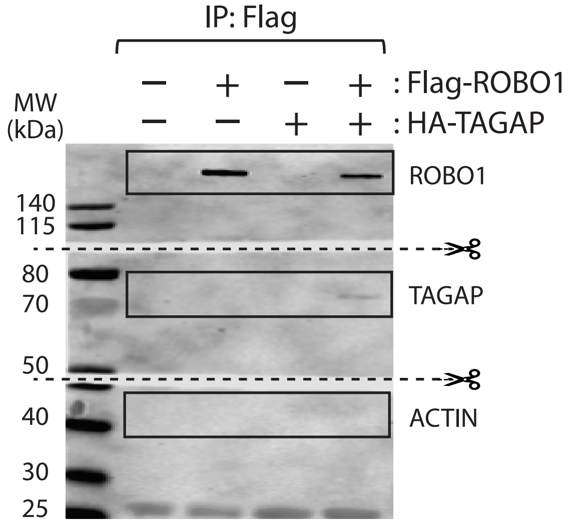

Figure 6H

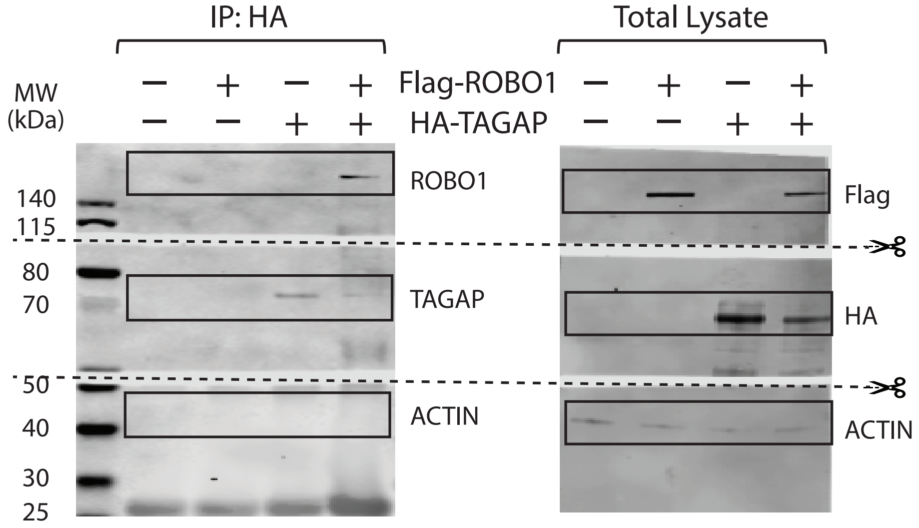

Supplement: Unedited blot and gel images [file jciinsight-11-203826-s180.pdf]
